# Use of web-based species occurrence information systems by academics and government professionals

**DOI:** 10.1371/journal.pone.0236556

**Published:** 2020-07-31

**Authors:** Elizabeth Martín-Mora, Shari Ellis, Lawrence M. Page

**Affiliations:** 1 School of Natural Resources and Environment, University of Florida, Gainesville, Florida, United States of America; 2 iDigBio and the Florida Museum of Natural History, University of Florida, Gainesville, Florida, United States of America; Universitat de Barcelona, SPAIN

## Abstract

Web-based information systems designed to increase access to species occurrence data for use in research and natural resource decision-making have become more prevalent over the past few decades. The effectiveness of these systems depends on their usability and extent of use by their intended audiences. We conducted an online survey of academics and government professionals in the United States to compare their species occurrence data needs and their perceptions and use of web-based species occurrence information systems. Our results indicate that although views and perceptions held by academics and government professionals about the importance, usefulness, and ease of use of these information systems tend to be similar, there were differences in their use of species occurrence data and web-based species occurrence information systems. The baseline information obtained in this study will help inform future directions for improvements in species occurrence information systems.

## Introduction

The Internet is often viewed as a medium that can facilitate access to and use of biodiversity data in research and natural resource decision-making [1–3]. The term biodiversity encompasses many levels of biological organization ranging from genomics to ecosystems, and biodiversity data includes species occurrence data, DNA barcode data, trait data, biotic interactions data, and many other data that describe the diversity of life on Earth [4]. Biodiversity data originate from multiple disciplines with different cultures and scientific approaches including their own taxonomic naming conventions and species concepts [5].

Species occurrence data, which are records on the occurrence (presence or absence) of an organism at a particular geographic location and time, are one of the most basic types of biodiversity data. These foundational data are used in research, management, and conservation of species [6]. Web-based information resources that provide species occurrence data are some of the most common types of biodiversity information resources. We defined for this study *web-based species occurrence information systems* as databases and their data retrieval systems (including applications software and application programming interfaces) provided via websites and the Internet to discover, access, visualize, download or summarize species occurrence data. As a subset of biodiversity information systems, these systems provide access to various types of species occurrence data, such as those originating from specimens in museum natural history collections, field observations made during research studies, observations made by segments of the general public, and signals from tracking devices among others. Although recent studies have analyzed publications to identify the type of research that has been conducted using species occurrence data from web-based information systems [7–9], considering the prevalence of these systems, it is surprising that little research has been conducted on users of these systems and their needs.

This study focuses on identifying and comparing the species occurrence data needs and use of web-based species occurrence information systems by academics and government professionals in the United States (U.S.). Since species occurrence data can be used for diverse purposes by varied user communities [6], web-based species occurrence information systems offer an opportunity for comparing system use and perspectives of user communities often mentioned as target audiences for biodiversity information systems. As use of an information system not only depends on system availability but also on preferences and perceptions of the user towards the system’s technology and its content (data) [10], to understand how user communities are using web-based species occurrence information systems, requires learning about the users data use processes, identifying the users preferred data types and sources, and identifying how users view or regard information systems. By examining similarities and differences on the use of web-based species occurrence information systems by users from different sectors of work, we are taking an initial step towards increasing our understanding on the needs of users and assessing how existing information systems may or may not be meeting those needs. Greater understanding about the needs of users of these systems could help inform development of new systems and improvements to existing ones.

### Background

Over the past 20 years, many have worked to make biodiversity data more broadly accessible via the Web [11–15]. Those leading the efforts to mobilize a variety of biodiversity data have ranged from individuals to institutions, have come from diverse disciplines (e.g. molecular biology, ecology, geology) and sectors (academia, government, non-profit), and have focused on different taxonomic groups of organisms and geographic scales [5]. In the absence of an overall strategy for making widely diverse data available, this diversity of data producers, disciplines, funding, geographic and taxonomic areas of focus, has contributed to a proliferation of online biodiversity information systems [16]. A 2019 study based on an analysis of publications from 2010–2017 identified 347 primary biodiversity databases/information systems across regional, national and global levels [8]. Examples of the diverse information systems for ecological and biodiversity data are provided by Michener [17] and Waide et al. [16] for the United States, and by Bingham et al. [18] for Europe. This variety of online biodiversity information systems reflect the needs and practices of the communities that produced them and their disciplines of origin.

Often, the intended audiences for web-based species occurrence information systems are scientists and natural resource professionals. Only a few studies have looked at academic users of species occurrence data and the information systems they use to obtain those data [19, 20]. Even less is known about non-academic users. In the early days of the Internet, natural resource professionals may not have considered the web an important resource for obtaining information needed for decision-making [21]. But in recent years, examples have emerged on how data from web-based information systems could be used by non-academic communities in planning, environmental impact assessments, and natural resource management [22, 23].

Understanding use of web-based species occurrence information systems requires learning about use of systems as well as data use practices. There is already a long tradition and large body of research about information use practices and information seeking behavior of academic scientists [24–28], academics overall [29, 30], and scientists in general [31]. These studies indicate that the information sources most often used by academics are peer-reviewed literature, personal communications, and web pages [24, 28, 29]. With the Internet, academics increasingly access peer-reviewed journals electronically [25], and use bibliographic databases and web search engines to find and obtain needed information [27]. Across disciplines, scientists appear to use similar sources to retrieve information, but the degree of use and information seeking behaviors may vary by discipline, type of research (basic versus applied), institution size, and certain other demographic characteristics [25, 26, 31].

Less is known about information seeking behaviors and information use practices of non-academic decision-makers and professionals in natural resource and science-related fields. But a few studies are providing some insights on information use practices of non-academic professionals in natural resource-related fields. Time constraints, insufficient searching skills, and reliance on professional networks have been found to influence the information seeking practices of natural resource professionals [32]. Davis et al. [20] reported academics and government professionals differ in their ease of finding biodiversity information and in the geographic scale of the biodiversity information used. Synthetic reviews and peer-reviewed literature were found to be important sources of information for decision-makers in California working on riparian conservation and restoration [21]. And web-based information systems were used by managers to access species occurrence data for management of invasive species in coastal wetlands of the Great Lakes [23].

In the past two decades, studies of data use by academic researchers have focused primarily on secondary use of data (data reuse) or use of data from others for a different purpose or study from that for which the data were originally collected [33–40]. Comparable information is lacking, however, on the reuse of data by non-research professionals in science and natural resources fields. Increased interest in data reuse stemmed in part from increased availability and access to electronic data and information resources via the Internet. Therefore, a study of use of data from web-based species occurrence information systems is really a study about data reuse. As data reuse is a type of data use, the term ‘reuse’ may confuse some readers, and in some instances it may be difficult to distinguish between data use and reuse [41]. We refer to data reuse as data use for the remainder of this publication.

Studies have found that social context, professional cultures and organizational structures have an effect on information use by professionals [42–45], while the user’s discipline, type of activity, personality, and experience with data, influence how they search for information [46]. In addition, aspects of the data, such as how and for what purposes data were produced within a discipline, appear to be important in generating trust to facilitate data use by researchers [47]. Because scientists and natural resource professionals tend to do different types of work, data use and their use of web-based species occurrence information systems might differ.

Use of digital data and electronic information systems that provide scientific data have been examined in the social sciences, health sciences, and a variety of natural, earth, and physical sciences [48–51]. In disciplines with well-developed digital infrastructures, such as the social sciences, researchers trust data from information systems because they have access to good documentation or know those who generated the data and data products [49, 51]. Not as much is known about use of web-based species occurrence information systems specifically, but studies on biodiversity data needs indicate users want systems that are easily discovered, and they want access to different types of data that are trusted and linked to other information resources [19, 20, 52].

### Present study

In this study we compared academics and government professionals in the United States on their species occurrence data needs, the sources they use for those data, and their perspectives on use of web-based species occurrence information systems as sources of species occurrence data. Level of information system use by participants was examined in terms of frequency and duration of system use, and percent of data they used that originated from those systems. This study aimed to answer the following questions:

- Who is using species occurrence data and web-based species occurrence information systems in their work?
- What type of species occurrence data do they use and how often?
- What sources do they use to obtain data and learn about existing web-based information systems?
- To what extent are they using web-based species occurrence information systems and data from those systems?
- What are their perceptions about web-based species occurrence information systems?
- What is their awareness of global and national species occurrence information systems?
- How likely are they to use a web-based species occurrence information system in the future?

Answers to these questions allow us to identify how academic and government professionals differ or are similar in their use of web-based species occurrence information systems and the data those systems provide, and what aspects of those systems need improvement in order to be more usable.

Next, we provide information about the methods used in this study, response and completion rates for our survey, and results of our research starting with a characterization of users of species occurrence data and web-based species occurrence information systems, followed by species occurrence data preferences, sources used to access species occurrence data and learn about existing information systems, use of web-based species occurrence information systems, perceptions about web-based species occurrence information systems, awareness of national and global web-based species occurrence information systems, and the likelihood of continued use of web-based species occurrence information systems.

## Methods

This study involved the voluntary participation of academic and government professionals. The use of human subjects in this study was conducted under a protocol reviewed and approved by the University of Florida Behavioral/NonMedical Institutional Review Board (IRB02).

### Questionnaire development

A questionnaire (see S1 Appendix) consisting of a combination of close-ended questions and open-ended questions was developed, with decisions about types of scales, number of points, polarity, use of a midpoint and a no-opinion option in Likert-type items based on a combination of questionnaire development recommendations provided in the peer-reviewed literature [53–56], general characteristics of the populations of interest, and potential responses for each specific Likert item question. Question content related to biodiversity data and information was based on findings in biodiversity data use [6, 19, 57] and the corresponding author’s work experience with biodiversity data and information. Questions about web-based information systems were based on prior findings from research on adoption of new technology and technology acceptance literature [10, 58–63] or to a lesser extent used in concept testing to evaluate and improve products [64, 65]. Questions about scientific data needs and use were based on findings in the information science literature [30, 36, 37, 66]. Closed-ended questions for the questionnaire were developed as individual Likert-type items rather than as part of Likert scales, and except for questions that included ‘select all that apply’ in the questionnaire (S1 Appendix), questions only allowed selection of one response.

The questionnaire was validated via an online pilot survey of 18 colleagues, 8 from academia and 10 from non-academia, who used species occurrence data and had agreed to take the survey to provide feedback about question clarity, appropriateness, and format. The questionnaire used in this study was finalized by including revisions based on feedback received from the pilot survey.

### Identification of potential participants

To identify academic institutions to sample, we selected universities categorized as ‘Doctoral Universities: Highest Research Activity’ in the Carnegie Classification of Institutions of Higher Education (CCIHE) [67]. To identify potential academic participants, we searched websites of academic departments at the selected universities for contact information of faculty and research scientists (S2 Appendix lists the academic fields considered relevant in this study).

To identify potential government participants, we obtained the names of state and federal government agencies from websites listing natural resource agencies in the U.S. More detail on the types of government agencies considered relevant for this study is provided in S2 Appendix. Websites of selected state agencies were identified in each of the 50 states of the U.S., while websites of relevant federal agencies were identified at national and regional/sub-regional office levels. The websites of relevant government agencies were searched for descriptions of programs that included species-related activities and the contact information (emails) of staff working on those programs.

This method identified a total of 3,154 faculty and research scientists (excluding students, adjunct and emeritus faculty, and short-term appointments) from academic institutions and a total of 4,250 staff from government agencies (3,195 from state government and 1,055 from federal government) for a total of 7,404 individuals. This large number of names with corresponding emails was gathered since sample size estimates for academics and government professionals, based on their population size [68] and a conservative 10% potential survey response rate, indicated we would need to invite 3,840 people to obtain a sample of participants with enough statistical power to detect an effect with a 0.05 probability. The number of potential invitees was doubled because we were interested in comparing academics and government professionals.

### Survey administration

Invitations to participate in an online survey were sent to 7,404 email addresses on March 2017 via the Qualtrics [69] software licensed to the University of Florida. An online informed consent was presented to potential participants, and those who agreed to participate in the study were given access to the survey. The online survey was open to potential participants for one month. The survey was opened again for one month in June 2017 to allow staff from a government agency that required agency approval to participate in the survey. Once approval from that agency was secured, a second invitation to participate in the survey was sent to 184 email addresses of agency staff who had received the initial invitation to the survey but had not responded.

Some invitees emailed the principal investigator indicating they wanted to forward the survey link to colleagues. A generic link to the online survey was created and provided to those who requested it. The online survey was permanently closed on July 2017.

### Data processing and analysis

This publication focuses on analysis and results from responses to close-ended questions of the survey only. Cleaning and processing of response records for some of the close-ended questions retained for analyses included re-categorizing some of the questions into fewer categories.

Descriptive statistics, as well as cross tabulations with sector of work, were generated for each closed-ended question using SPSS [70]. Weights were not applied to samples since the majority of analyses conducted were nonparametric. To compare responses between academic and non-academic sectors, the following statistical tests were conducted:

- Chi-square test of independence—A chi-square test of independence is a nonparametric test to determine whether two or more categorical variables are independent or are associated with each other. The test assumes random samples, independent observed data provided as counts or frequencies, mutually exclusive categorical variables with data that are nominal or ordinal, and eighty percent of expected (computed) cell frequencies should be greater than five and none should be less than one [71, 72]. Limitations of the test include not being appropriate for continuous data, a need for large samples, lack of specificity of results about the direction or strength of the relationship between variables, and no causal relationship [72, 73]. Chi-square tests of independence were used in our study to analyze nominal variables and also variables that were ordinal but had less than five categories. Data used in our chi-square analyses were percent frequencies.
- Mann-Whitney U test—Mann-Whitney U test is a nonparametric test that compares distributions of a dependent variable for two groups to determine if they are from the same population. That test assumes random samples, the groups to be independent, continuous or ordinal data, equal group variances, and similar shape for distribution of each group if reporting the difference as medians [74]. Test limitations include having less statistical power and being more sensitive to non-homogeneous variances when compared to a t-test [74]. The Mann-Whitney U test appears to be more powerful than a t-test when skewed data and outliers occur as well as with unequal sample sizes [74–76]. At large sample sizes the test is approximated to a normal distribution. Mann-Whitney U tests were used in our study to analyze ordinal variables with five or more categories, and continuous variables that were not normally distributed. Since a Mann-Whitney U test compares mean ranks of two samples to determine if they came from the same distribution, results from Mann-Whitney U tests conducted in this study are based on mean ranks. However, in addition to medians as measures of central tendency, means of ordinal variables are also provided given that the number of categories for each ordinal variable is somewhat small, and a mean allows for easier understanding of results from inferential statistics on Likert-type item scaled variables.
- Independent samples t-test—A t-test for independent samples is a parametric test that compares the means of two independent groups. The t-test assumes random samples, a continuous dependent variable and an independent categorical variable, a dependent variable that is normally distributed, and equal group variances [77, 78]. Test limitations include sensitivity to sample sizes, being less robust to violations of the equal variance and normality assumptions when sample sizes are unequal [75] and performing better with large sample sizes [79]. T-tests were used in our study to compare means between groups for continuous variables.
- Spearman’s Rank-Order Correlation—The Spearman’s rank-order correlation is a nonparametric test that determines whether there is an association (correlation) between two variables. The test assumes independent observations, continuous or ordinal data, and a monotonic relationship between the variables [80, 81]. Limitations of Spearman’s rank-order correlation include low power with small sample sizes, and it is considered less powerful than Pearson’s correlation when distributions are normal [80–82]. Spearman’s rank-order correlation test was used in our study to identify correlations between two ordinal variables.

All chi-square tests, Mann-Whitney U tests, t-tests, and Spearman’s correlation analyses were evaluated for significance as two-tailed tests with an alpha of 0.05. For statistically significant chi-square tests, pairwise comparisons between academic and government participants where conducted on each class of a variable using adjusted standardized residuals as z-scores and correcting the critical value by the number of comparison tests resulting in a Bonferroni adjustment to the critical value of alpha.

## Results

### Survey response and completion rates

At the close of the survey, 1,011 of the 7,404 individuals invited to participate had responded. Most responses came from direct invitations (94%), with only a small percentage (6%) coming from the generic link to the survey. For a participant’s response to be included in the dataset that was analyzed, it had to at a minimum reach the point in the questionnaire where the participant provided a response to question Q3.3 ‘Which of the following best describes your use of species occurrence data?’ (S1 Appendix). Of recorded responses, 942 had included an answer to Q3.3, and therefore had sufficient information to be retained for analyses on use of species occurrence data and web-based species occurrence information systems. The completion rate (calculated from those who completed the survey given they had started it) of this survey was 95%, while the response rate (calculated from those who completed the survey out of the total number of invitees) of this survey was 15%. The response rate of 15% is within the range of what is average (10–20%) for academic staff or professionals when invited to participate in unsolicited external online surveys without incentives [83, 84]. S3 Appendix lists the disposition codes of the online survey used to classify responses of those sampled and the equations for completion rate and response rate calculations adopted from the American Association for Public Opinion Research [85].

In analyses that follow, the number of responses is provided for each survey question. Since participants could skip questions, the number of responses varies by question.

### Users of species occurrence data and web-based species occurrence information systems

#### Participants’ sector of work

Of the 941 survey participants who reported their *Sector of Work*, 35% (n = 329) were from *academia*, 44% (n = 411) from *state government*, 20% (n = 189) from *federal government*, 1% (n = 11) from *non-profit organizations*, and less than 1% (n = 1) from a *for-profit business*. Non-academic participants were for the most part professionals who worked for government, but there were a few participants who had indicated working for non-profit organizations or a for-profit business. Even though we did not directly recruit participants from non-profit organizations or for-profit businesses, it is possible those participants may have collaborated or worked under contract or agreements with government agencies. The responses they provided were similar to those who were employed in government, and hence responses from those participants were grouped with those from state and federal government participants into a summarized government sector of work consisting of government professionals. The summarized sectors of work of 35% (n = 329) academics and 65% (n = 612) government professionals were used in subsequent data analyses of this study.

### Demographic characteristics of survey participants

The demographic characteristics of participants are listed in Table 1. In terms of *Gender*, there were more male than female respondents for both academia and government sectors. These percentages did not differ significantly between the two sectors of work (Table 1), but the proportions of males and females within each sector were significantly different (P<0.001) from an expected probability of 0.50 for each gender based on results of a Binomial test.

**Table 1:** Demographic characteristics of survey participants.

Variable	Category (code)	Sector of Work		Chi-square test of independence
		Academia % Frequency	Government % Frequency	
Gender	Female	31	39	χ^2^ = 5.452
	Male	69	61	df = 2
	Other	0	0	P = 0.065
	*Number participants (n)*	*(268)*	*(519)*	n = 787
Age***	18–30 years old (1)	3	5	
	31–40 years old (2)	27	26	
	41–50 years old (3)	27	30	χ^2^ = 28.193
	51–60 years old (4) †	19	27	df = 5
	61–70 years old (5) †	19	12	P < 0.001
	71–80 years old (6) †	4	1	n = 928
	81 or more years old (7)	0	0	
	*Number participants (n)*	*(324)*	*(604)*	
Year of Last Degree***	2011 or later (1)	17	12	
	2001–2010 (2)	30	35	
	1991–2000 (3) †	22	29	χ^2^ = 27.693
	1981–1990 (4)	19	19	df = 5
	1971–1980 (5) †	10	5	P < 0.001
	1961–1970 (6) †	2	0	n = 939
	Prior to 1960 (7)	0	0	
	*Number participants (n)*	*(329)*	*(610)*	
Highest Level of Education***	High school graduate	0	0	
	Trade / technical / vocational training	0	0	
	Some college, no degree	0	0	χ^2^ = 443.898
	Associate degree	0	0	df = 4
	Bachelor’s degree †	1	25	P < 0.001
	Master’s degree †	3	50	n = 941
	Doctorate degree †	96	25	
	*Number participants (n)*	(329)	(612)	
Region of Residence	Northeast	14	10	
	Midwest	22	28	χ^2^ = 5.241
	South	37	35	df = 3
	West	28	27	P = 0.155
	*Number participants (n)*	*(297)*	*(556)*	n = 853

*** Statistically significant at P ≤ 0.001 (Chi-square tests).

^†^ Statistically significant z-score with Bonferroni correction P ≤ 0.05 for multiple comparisons.

For *Age* (Table 1), the greatest number of academic participants were in the categories of *41–50* years and *31–40* years. For government participants, the greatest number of respondents were in three categories: *41–50*, *51–60* and *31–40* years. The result from a Mann-Whitney U Test on *Age* (Table 2) was borderline non-significant when comparing ranked distributions of *Age* between academics and government professionals. Even though there were no differences in the measures of central tendency for *Age*, a chi-square test on *Age* (Table 1) indicated there were higher percentages of participants from academia in the age categories of 51 years and older.

**Table 2:** Mann-Whitney U tests of ordinal demographic variables.

Variable	U Statistic (Z)	P-value	Academia			Government		
			Number	Mean (SD)	Median (IQR)	Number	Mean (SD)	Median (IQR)
Age	90490.5 (-1.950)	0.051	324	3.4 (1.3)	3 (2)	604	3.2 (1.1)	3 (2)
Year of Last Degree	97235.5 (-0.811)	0.417	329	2.8 (1.3)	3 (2)	610	2.7 (1.1)	3 (1)

For *Year of Last Degree* received, the highest percentages of both academic and government participants had received their last degrees during the periods *2001–2010* and *1991–2000* (Table 1). Overall, there was no significant difference in the mean ranks and ranked distribution for *Year of Last Degree* between academic and government participants based on a Mann-Whitney U test (Table 2). However, there were differences between the two groups in terms of specific categories in the scale used for this variable. Those with the longest time since *Year of Last Degree* (periods *1971–1980* and *1961–1970*) were more likely to be academics than from government (Table 1).

Spearman’s correlation analyses show there is a strong positive correlation between *Age* and *Year of Last Degree* across sectors (r_s_ = 0.824, P < 0.001, n = 929) and within each sector (academia: r_s_ = 0.872, P < 0.001, n = 324; government: r_s_ = 0.794, P < 0.001, n = 604). Given the high correlation, similar distributions of *Age* and *Year of Last Degree*, and slight differences in *Age* for the older age categories between academics and government professionals, we selected *Age* as the best indicator of participants’ level of experience in their field of work.

The *Highest Level of Education* attained by the majority of academic participants was a *doctorate degree* (Table 1). Government participants were more diverse in terms of their *Highest Level of Education*. Half of government participants held a *master’s degree* as their highest degree, while the remainder were equally split between a *doctorate degree* and *bachelor’s degree*. Overall, the academic and government sectors were significantly different in terms of their *Highest Level of Education* (Table 1), with the percentage of respondents at the three highest levels of education (*bachelor’s degree*, *master’s degree* and *doctorate degree*) being significantly different between academics and government professionals based on their z-scores with a Bonferroni correction for multiple comparisons. Survey participants who held a bachelor’s or master’s degree were almost solely from government, while the greatest percentage of participants who held doctorate degrees were from academia.

We analyzed the geographic distribution (*Region of Residence*) of survey participants as defined by the United States Census Bureau [86] and found the greatest percentage of academic participants resided in the *South*, followed by the *West*; the majority of government participants also resided in the *South*, followed by the *Midwest* and the *West* (Table 1). Overall, there was no significant difference in *Region of Residence* between academic and government participants in our survey.

### Scope of work of survey participants

Table 3 provides a summary of the participants’ *Primary Area of Work*. Original categories for *Primary Area of Work* are those that were included in the questionnaire and were selected by participants in their survey responses. Because some of the original categories had very low percent frequencies, the principal investigator derived final categories of *Primary Area of Work* by grouping original categories into a smaller number to facilitate their use in analyses. The majority of academic participants indicated they work primarily on *scientific research* and to a lesser extent on *teaching/education*, with only a few academics working in *natural resource professions* (Table 3 final categories). The majority of government participants reported working primarily in *natural resource professions* and to a lesser extent *scientific research*, with only few government professionals in *teaching/education* (Table 3 final categories). Overall, there was a significant association between *Primary Area of Work* and *Sector of Work*, with percent frequencies for the three areas of work (*scientific research*, *natural resource professions*, and *teaching/education*) being significantly different between academic and government participants based on Z-scores from standardized adjusted residuals with a Bonferroni correction for multiple comparisons (Table 3).

**Table 3:** Primary area of work of participants.

Variable	Category	Sector of Work	
		Academia % Frequency	Government % Frequency
Original categories—Primary Area of Work	Scientific research	86	20
	Teaching and education	11	2
	Natural resource management & conservation practice	1	63
	Environmental planning	0	6
	Policy / administration	0	5
	Information transfer / communication	1	5
	*Number participants (n)*	*(329)*	*(612)*
Final categories—Primary Area of Work ***	Scientific research †	86	20
	Teaching and education †	11	2
	Natural resource professions †	3	79
	*Number participants (n)*	*(329)*	*(612)*

*** Statistically significant at P ≤ 0.001 (Chi-square test).

^†^ z-score Bonferroni correction P ≤ 0.05.

Chi-square test of independence for Final categories—Primary Area of Work: χ^2^ = 490.747, df = 2, P < 0.001, n = 941.

Most participants, whether from academia (n = 275) or government (n = 120), who indicated their *Primary Area of Work* was *scientific research* reported they worked in the *biological sciences* (81% of academics vs. 75% of government professionals), followed distantly by *multidisciplinary or applied sciences* (11% of academics vs. 17% of government professionals), with the remaining working in the *physical sciences*, *mathematics and statistics*, and *other disciplines*. Except for *mathematics and statistics* (χ^2^ = 9.703, df = 4, P = 0.046, n = 395), which was represented by a higher percent of government professionals than academics, the scientific research disciplines of academic and government respondents were quite similar.

Slightly more than half (55%) of government participants (n = 369) who identified their original *Primary Area of Work* as *natural resource management and conservation practice*, indicated they worked on *species management and conservation*. The remaining worked on *land/water management & conservation* (30%), *nuisance species/invasive species control and management* (8%), and *other* types of work (7%).

The *Geographic Scope of Work* of academics was primarily at *national* and *international* levels, while that of government professionals was primarily at the *state* level, followed distantly by a *regional* focus within the U.S. (Table 4). Chi-square tests of independence for individual geographic level with sector of work (academia and government) showed the following significant differences between sectors: *local*, *sub-region within a state*, *state*, *regional*, *national*, *international*, and *other* (Table 4). These results demonstrate that *Geographic Scope of Work* is dependent on *Sector of Work* (i.e., academia or government).

**Table 4:** Geographic scope of work of participants.

Geographic scope of work ^a^	Sector of Work		Chi-square test of independence
	Academia % Frequency	Government % Frequency	
Local *	10	6	χ^2^ = 4.66 df = 1 P = 0.031 n = 940
Sub-region within a state *	10	16	χ^2^ = 6.366 df = 1 P = 0.012 n = 940
State ***	21	57	χ^2^ = 111.219 df = 1 P < 0.001 n = 940
Regional (within U.S.) **	35	26	χ^2^ = 9.148 df = 1 P = 0.002 n = 940
National ***	47	17	χ^2^ = 93.576 df = 1 P < 0.001 n = 940
International ***	43	7	χ^2^ = 181.231 df = 1 P < 0.001 n = 940
Other *	5	2	χ^2^ = 4.375 df = 1 P = 0.036 n = 940
*Number participants (n)*	*(329)*	*(611)*	

^a^ ‘select all that apply’ type of question. Percentages do not add to 100%.

* Statistically significant at P ≤ 0.05.

** Statistically significant at P ≤ 0.01.

*** Statistically significant at P ≤ 0.001.

### Use of species occurrence data

**Fig 1:**
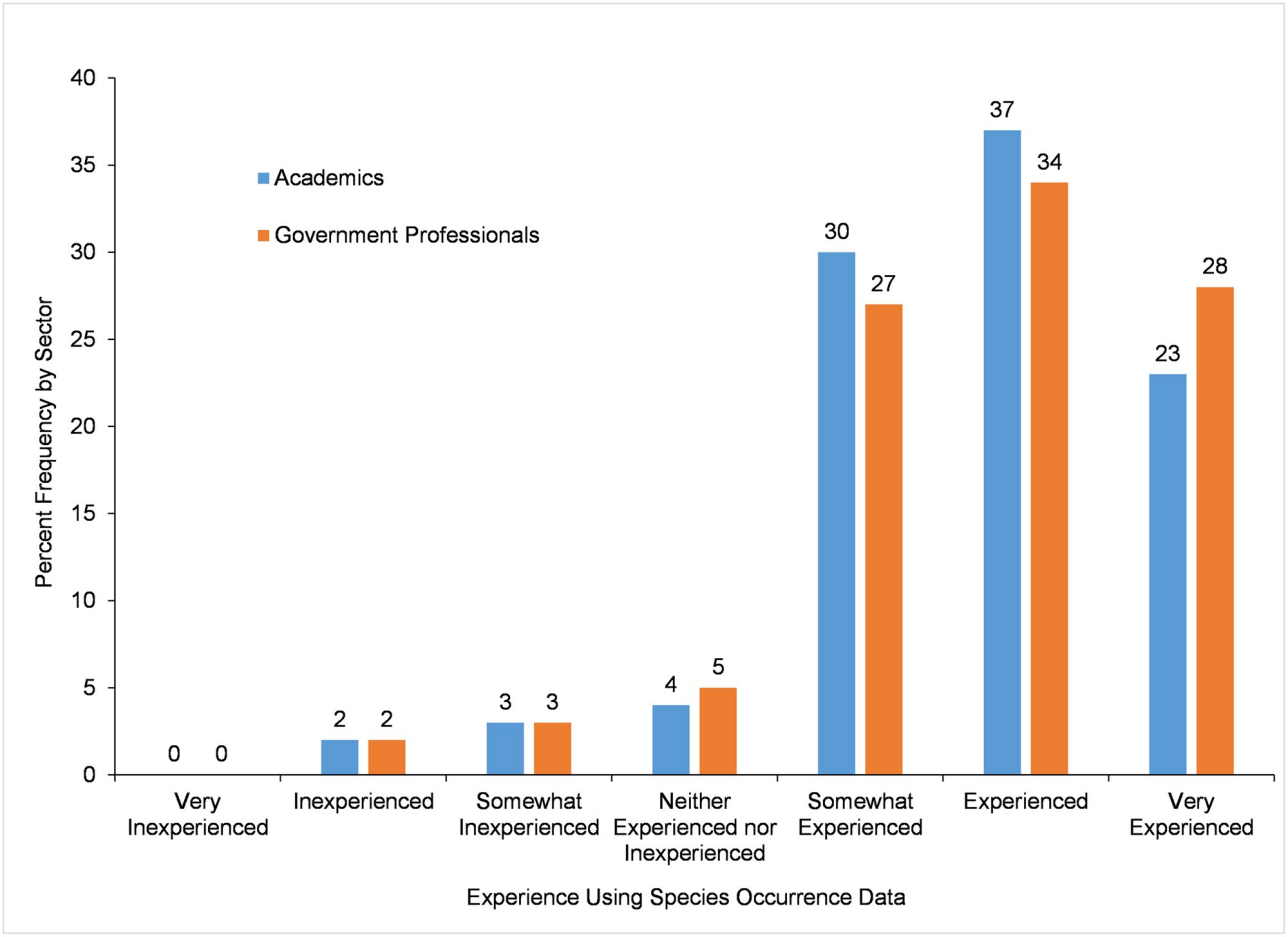
Level of experience using species occurrence data.

Participants were asked to self-report their level of *Experience Using Species Occurrence Data* (Fig 1). Most participants reported being at least *somewhat experienced* in using species occurrence data. The ranked distributions and mean ranks of self-reported level of experience were not significantly different between academic and government participants (Table 5).

**Table 5:** Mann-Whitney U tests of level of experience using species occurrence data.

Variable	U Statistic (Z)	P-value	Academia			Government		
			Number	Mean (SD)	Median (IQR)	Number	Mean (SD)	Median (IQR)
Experience Using Species Occurrence Data	95805.000	0.358	326	5.7 (1.1)	6 (1)	609	5.7 (1.2)	6 (2)

The survey also asked participants about their use of data originating from other people or programs beyond their own. There was no difference in the *Use of Species Occurrence Data from Others* between academic and government participants, with 92% from each sector of work having used species occurrence data from others in their work (χ^2^ = 0.050, df = 1, P = 0.824, n = 939).

**Fig 2:**
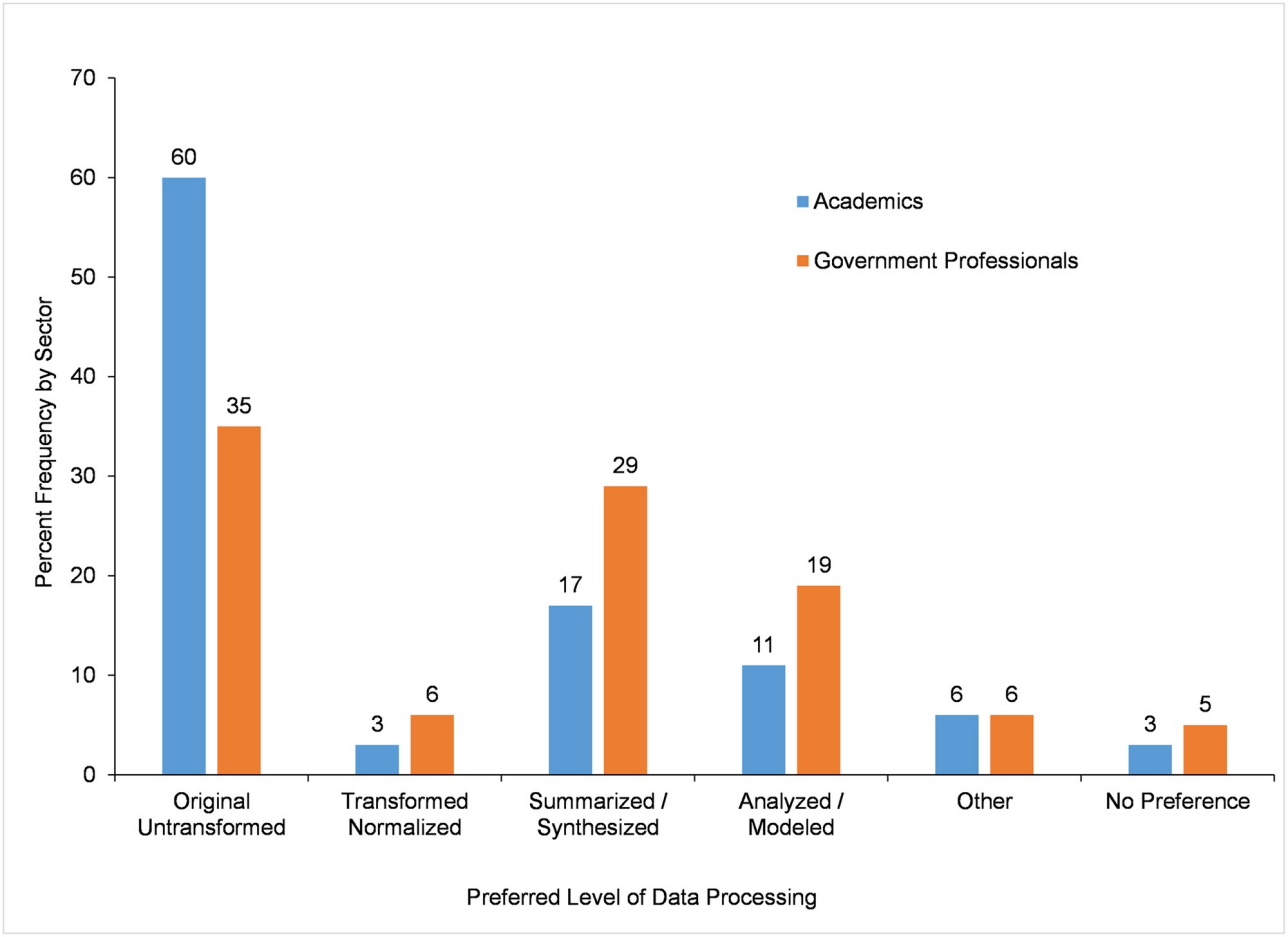
Preferred level of species occurrence data processing for use.

Survey participants were asked what kind of species occurrence data (*Preferred Level of Data Processing*) they preferred (Fig 2). Academic participants (n = 300) preferred by far *original untransformed data* (60%). The *Preferred Level of Data Processing* of government participants (564 participants) was more evenly distributed across categories, with the greatest preferences for *original untransformed* data (35%) and *summarized/synthesized* data (29%). Overall, there is a statistically significant relationship between *Preferred Level of Data Processing* and *Sector of Work* (χ^2^ = 52.444, df = 5, P < 0.001, n = 864). Examination of z-scores of pairwise comparisons with a Bonferroni adjustment indicate that the percent frequency of respondents who selected *original untransformed* data, *summarized/synthesized* data, and *analyzed/modeled* data was significantly different between academic and government participants. The greatest difference and contribution to the significance of the chi-square test was from *original untransformed* data (59.7% academics vs. 34.6% government professionals), followed by *summarized/synthesized* data (17.0% academics vs. 28.7% government professionals), and then by *analyzed/modeled* data (11.0% academics vs. 19.1% government professionals).

The preference of government professionals for two levels of data processing might be explained by the work they do. To examine if that might be the case in this study, we identified the primary area of work of government participants who had indicated they preferred *original untransformed* data or *summarized/synthesized* data. The 195 government participants who selected a preference for *original untransformed* data worked primarily in natural resource professions (68%) and scientific research (32%). Nearly all (88%) of the 162 government participants who selected a preference for *summarized/ synthesized* data worked primarily in natural resource professions. Therefore, it appears that even though there is a higher percentage of government professionals who work in scientific research that prefer *original untransformed* data when compared to those participants who prefer *summarized/synthesized* data, the greatest percentages of those government professionals who prefer *original untransformed* data are working in natural resource professions rather than in scientific research.

Survey participants were asked about their use of different types of species occurrence data. Table 6 shows the percentage of participants who reported using each of the following types of data: *Observational Data* (observational data from inventories, surveys, and monitoring programs), *Specimen Data* (data from specimens in natural history collections), *Instrument Data* (data from images, recordings, and instruments such as remote sensing and DNA sequencing), *Citizen Science Data* (data from citizen science programs), and *Species Ranges and Distributions* (species geographic ranges and distributions). These results show that the highest use by both sectors were for *Observational Data* and *Species Ranges and Distributions*. Government professionals significantly used *Observational Data* and *Species Ranges and Distributions* more than academics. And while academics significantly used more *Specimen Data* than government professionals, the latter used more *Citizen Science Data* than academics, although that use was low when compared to use of other data types (Table 6). Only for *Instrument Data* was the percent frequency of use similar for the two sectors of work.

**Table 6:** Frequency of use of different types of species occurrence data.

Data Type	Sector of Work		Chi-square test of independence
	Academia % Frequency(n)	Government % Frequency (n)	
Observational Data ***	87.4 (294)	98.0 (555)	χ^2^ = 40.508 df = 1 P < 0.001 n = 849
Specimen Data ***	63.4 (292)	45.7 (545)	χ^2^ = 23.773 df = 1 P < 0.001 n = 837
Instrument Data	67.0 (288)	66.7 (544)	χ^2^ = 0.007 df = 1 P = 0.934 n = 832
Citizen Science Data ***	44.3 (289)	62.0 (547)	χ^2^ = 23.983 df = 1 P < 0.001 n = 836
Species Ranges and Distributions *	86.4 (294)	91.3 (554)	χ^2^ = 5.042 df = 1 P = 0.025 n = 848

* Statistically significant at P ≤ 0.05.

*** Statistically significant at P ≤ 0.001.

**Fig 3:**
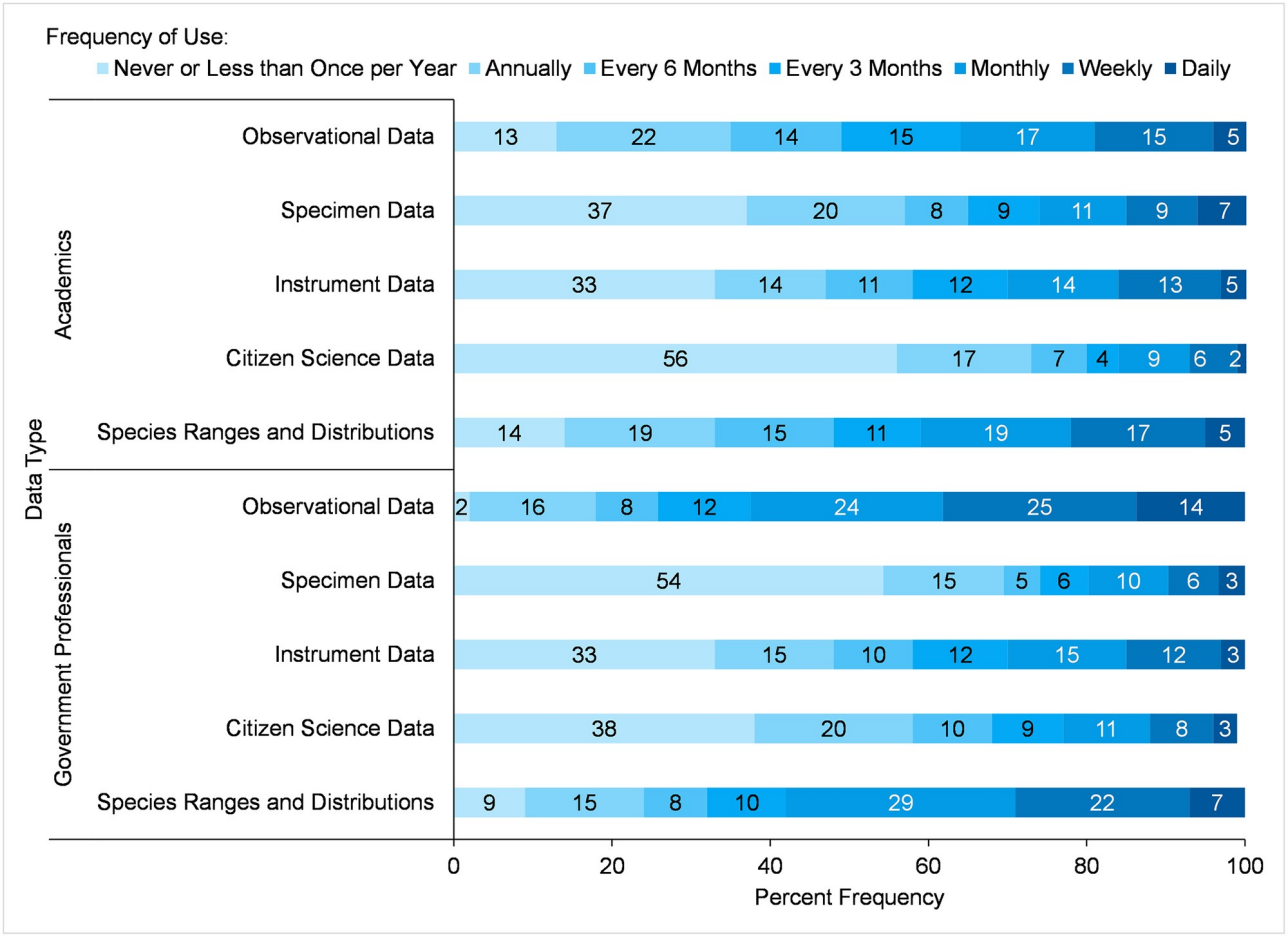
Frequency of use of species occurrence data types by academic and government participants.

Survey participants provided responses about their frequency of use of the different types of species occurrence data along a scale ranging from *daily*, *weekly*, *monthly*, *every 3 months*, *every 6 months*, *annually*, and *never or less than once per year* (Fig 3). The types of data used the least (*never or less than once a year*) by academic participants were *Citizen Science Data* (56%), *Specimen Data* (37%), and *Instrument Data* (33%). For government participants, although the same types of data were used the least, the percent frequencies were reversed, with government participants not using *Specimen Data* (54%) the most, followed by *Citizen Science Data* (38%), and *Instrument Data* (33%). The highest percentages of responses from academic participants for any one category of frequency of use were *annual* use of *Observational Data* (22%), *annual* use of *Specimen Data* (20%), and *annual* (19%) and *monthly* (19%) use of *Species Ranges and Distributions*. For government participants, the highest percentages for any one category were *monthly* (29%) and *weekly* (22%) use of *Species Ranges and Distributions*, and *weekly* (25%) and *monthly* (24%) use of *Observational Data*. Comparing the frequency of use categories for each type of data between academic and government participants with Mann-Whitney U tests (Table 7), showed there were significant differences between academic and government participants in mean ranks frequency of use of *Observational Data*, *Species Ranges and Distributions*, *Specimen Data*, and *Citizen Science Data*. Mean ranks of use of *Instrument Data* were similar for academics and government professionals. These results of measures of central tendency for ordinal categories of frequency of use are similar to those obtained from the chi-square tests of the overall percentage of participants who used a particular data type.

**Table 7:** Mann-Whitney U tests of frequency of use of different types of species occurrence data.

Data Type	U Statistic (Z)	P-value	Academia			Government		
			Number	Mean (SD)	Median (IQR)	Number	Mean (SD)	Median (IQR)
Observational Data ***	55914.5 (-7.667)	< 0.001	294	3.7 (1.8)	4 (3)	555	4.7 (1.7)	5 (3)
Specimen Data ***	65175.0 (-4.600)	< 0.001	292	2.9 (2.0)	2 (4)	545	2.4 (1.9)	1 (3)
Instrument Data	76914.0 (-0.442)	0.659	288	3.2 (2.0)	3 (4)	544	3.1 (1.9)	3 (4)
Citizen Science Data ***	64111.0 (-4.728)	< 0.001	289	2.2 (1.7)	1 (2)	547	2.7 (1.9)	2 (3)
Species Ranges and Distributions ***	66995.0 (-4.328)	< 0.001	294	3.7 (1.8)	4 (3)	554	4.3 (1.8)	5 (3)

Category codes: *never or less than once per year* (1), *annually* (2), *every 6 months* (3), *every 3 months* (4), *monthly* (5), *weekly* (6), *daily* (7).

*** Statistically significant at P ≤ 0.001.

### Sources of data and information

Survey participants who used species occurrence data were asked about use of five sources of species occurrence data *(Data Source)*: *Colleagues* (colleagues and personal contacts), *Reports* (agency reports, assessment / planning documents), *Books*, *Publications* (articles in trade publications and peer-reviewed journals), and the *Web* (web-based information resources and systems). The *Web* in this question of the survey had a broad scope and included web-based information resources such as websites and web-based search engines, in addition to web-based information systems on biodiversity and species occurrences. The results listed in Table 8 on percentage of participants who reported using the various data sources indicate that academic participants use primarily the *Web*, followed by *Publications*, and then *Colleagues* to obtain data from others, while government participants use primarily *Colleagues*, *Reports* and the *Web* as sources of data from others. There was a significant difference between academics and government professionals in their overall use of *Colleagues* and *Reports* as sources of data, while the slight difference in percent frequency of use of *Books* between academic and government participants was borderline non-significant (Table 8). The use of *Publications* and use of the *Web* were similar between academic and government participants.

**Table 8:** Frequency of use of sources of species occurrence data.

Data Source	Sector of Work		Chi-square test of independence
	Academia % Frequency (n)	Government % Frequency (n)	
Colleagues ***	79.7 (290)	92.0 (550)	χ^2^ = 26.897 df = 1 P < 0.001 n = 840
Reports ***	65.2 (290)	91.7 (552)	χ^2^ = 92.608 df = 1 P < 0.001 n = 842
Books	59.1 (286)	65.9 (540)	χ^2^ = 3.771 df = 1 P = 0.052 n = 826
Publications	80.5 (287)	75.2 (544)	χ^2^ = 2.986 df = 1 P = 0.084 n = 831
Web	88.7 (292)	89.7 (554)	χ^2^ = 0.206 df = 1 P = 0.650 n = 846

*** Statistically significant at P ≤ 0.001.

**Fig 4:**
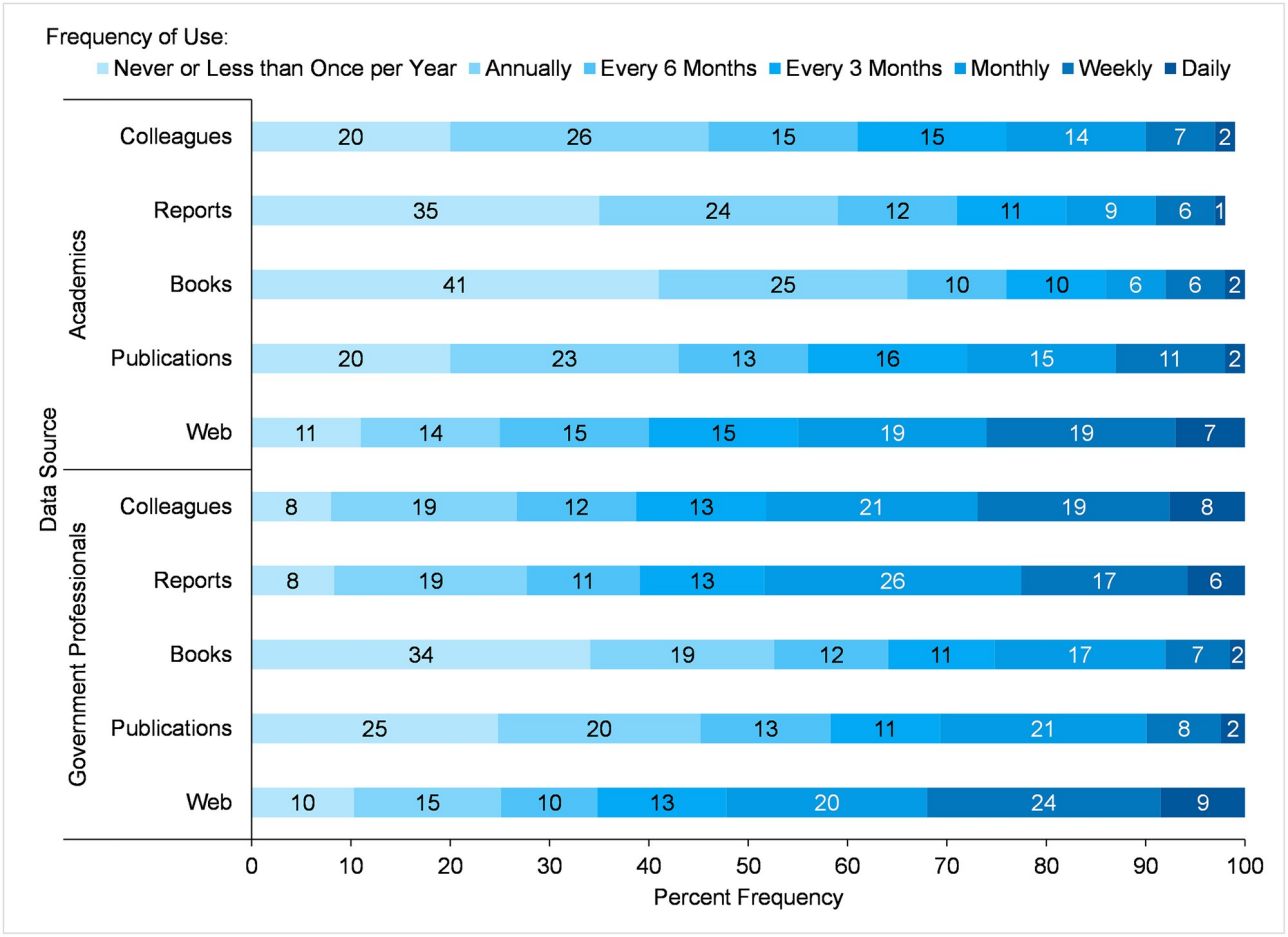
Frequency of use of data sources by academic and government participants who used species occurrence data.

Survey participants who used species occurrence data indicated their frequency of use, ranging from *daily*, *weekly*, *monthly*, *every 3 months*, *every 6 months*, *annually*, and *never or less than once per year*, for different sources of species occurrence data (Fig 4). This group of participants included those who did not use web-based species occurrence information systems. The highest percentage of any one frequency category of use of a data source by academic participants was *annually* for *Colleagues* (26%), *annually* for *Books* (25%) and *annually* for *Reports* (24%), while for government participants, it was *monthly* for *Reports* (26%) and *weekly* for the *Web* (24%). The least used (*never or less than once per year*) sources of data for academic participants were *Books* (41%) and *Reports* (35%), while for government participants were *Books* (34%) and *Publications* (25%). A comparison between sectors with Mann-Whitney U tests (Table 9) when considering all categories, showed there was a significant difference in the mean ranks of frequency of use between academics and government professionals for *Colleagues*, *Reports*, and *Books*. Academics and government professionals were similar in the mean ranks of frequency of use for *Publications* and the *Web* (Table 9). Therefore, results obtained from tests of measures of central tendency are similar to those obtained from chi-square tests of overall percentage of participants’ frequency of use of a data source.

**Table 9:** Mann-Whitney U tests of frequency of use of different sources of species occurrence data.

Data Source	U Statistic (Z)	P-value	Academia			Government		
			Number	Mean (SD)	Median (IQR)	Number	Mean (SD)	Median (IQR)
Colleagues ***	54582.5 (-7.629)	< 0.001	290	3.1 (1.7)	3 (2)	550	4.1 (1.8)	4 (4)
Reports ***	44597.5 (-10.724)	< 0.001	290	2.6 (1.7)	2 (3)	552	4.0 (1.8)	4 (3)
Books ***	66724.0 (-3.323)	< 0.001	286	2.4 (1.6)	2 (2)	540	2.8 (1.8)	2 (4)
Publications	74606.0 (-1.069)	0.285	287	3.3 (1.7)	3 (3)	544	3.2 (1.8)	3 (3)
Web	74772.0 (-1.834)	0.067	292	4.0 (1.8)	4 (4)	554	4.2 (1.9)	5 (4)

Category codes: *never or less than once per year* (1), *annually* (2), *every 6 months* (3), *every 3 months* (4), *monthly* (5), *weekly* (6), *daily* (7).

*** Statistically significant at P ≤ 0.001.

**Fig 5:**
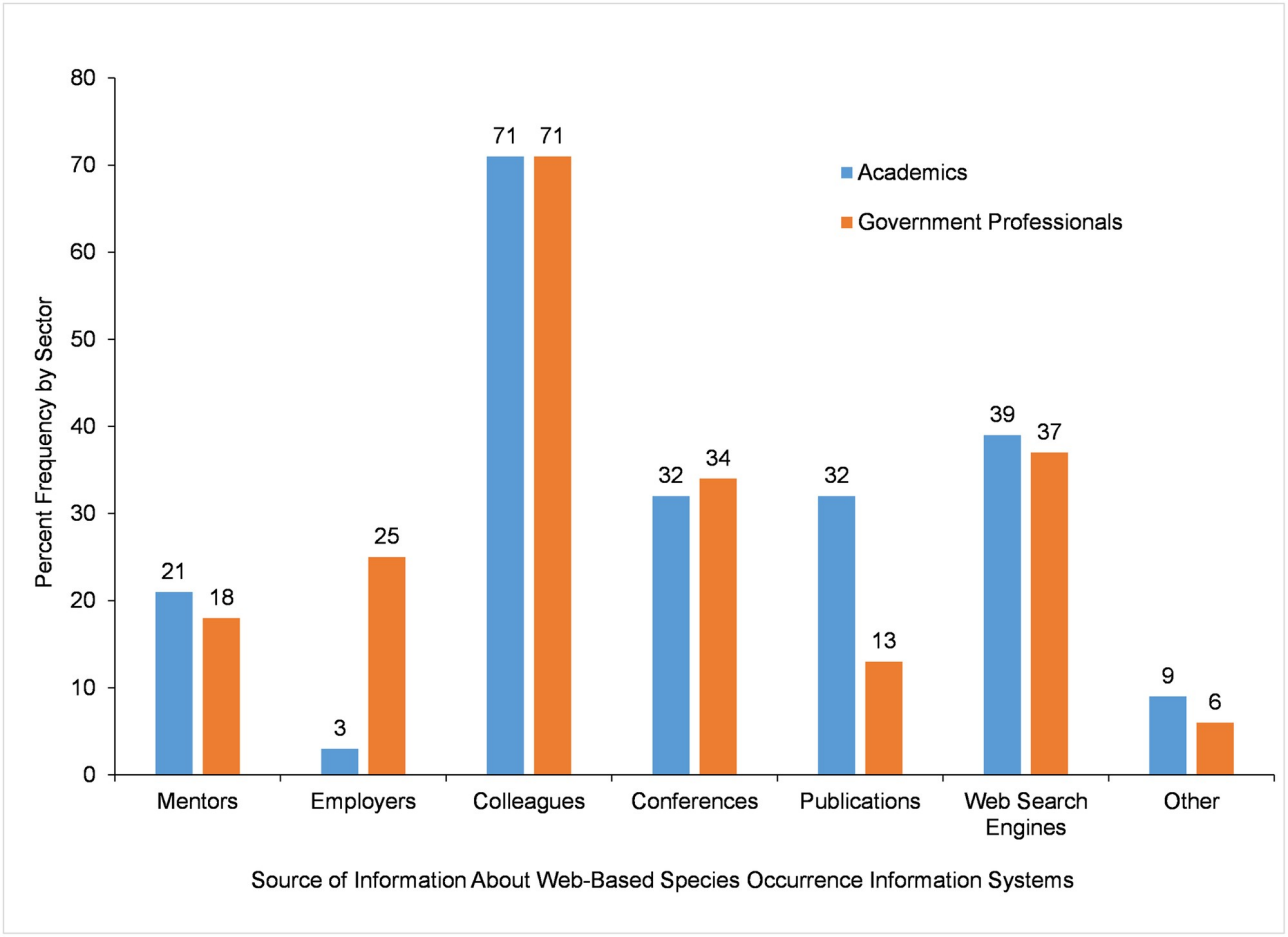
Source of information where participants learned about web-based species occurrence information systems.

Across both sectors, of those who had used a web-based species occurrence information system in the last 12 months, the majority (71%) learned about the existence of these information systems from *Colleagues* (Fig 5). To a much lesser extent but with similar percentages for both sectors, academic and government participants also learned about information systems via *Web Search Engines*, *Conferences*, *Mentors*, and *Other* (Fig 5). The only statistically significant differences between sectors were for *Employers* and *Publications* (Table 10). Government participants were more likely to learn about web-based species occurrence information systems from *Employers* than academics, while academic participants were more likely to learn about these information systems via *Publications* than government participants (Table 10).

**Table 10:** Frequency of source of information where participants learned about web-based species occurrence information systems.

Source of information	Sector of Work		Chi-square test of independence
	Academia % Frequency	Government % Frequency	
Mentors	21.3	17.6	χ^2^ = 1.414 df = 1 P = 0.234 n = 720
Employers ***	2.9	25.0	χ^2^ = 54.724 df = 1 P < 0.001 n = 720
Colleagues	71.3	71.4	χ^2^ = 0.001 df = 1 P = 0.974 n = 720
Conferences	32.4	34.5	χ^2^ = 0.311 df = 1 P = 0.577 n = 720
Publications ***	31.6	13.4	χ^2^ = 33.602 df = 1 P < 0.001 n = 720
Web Search Engines	39.3	36.6	χ^2^ = 0.536 df = 1 P = 0.464 n = 720
Other	9.4	5.7	χ^2^ = 3.518 df = 1 P = 0.061 n = 720
*Number participants (n)*	*(244)*	*(476)*	

*** Statistically significant at P ≤ 0.001.

### Use of web-based species occurrence information systems

Survey respondents were asked if they had used at least one web-based species occurrence information system in the last 12 months (*Use of Web-based species occurrence information systems in the last 12 months*). Those who answered *Yes*, 76% of academic participants (n = 323) and 79% of government participants (n = 607) were asked more questions about their use of these systems.

**Fig 6:**
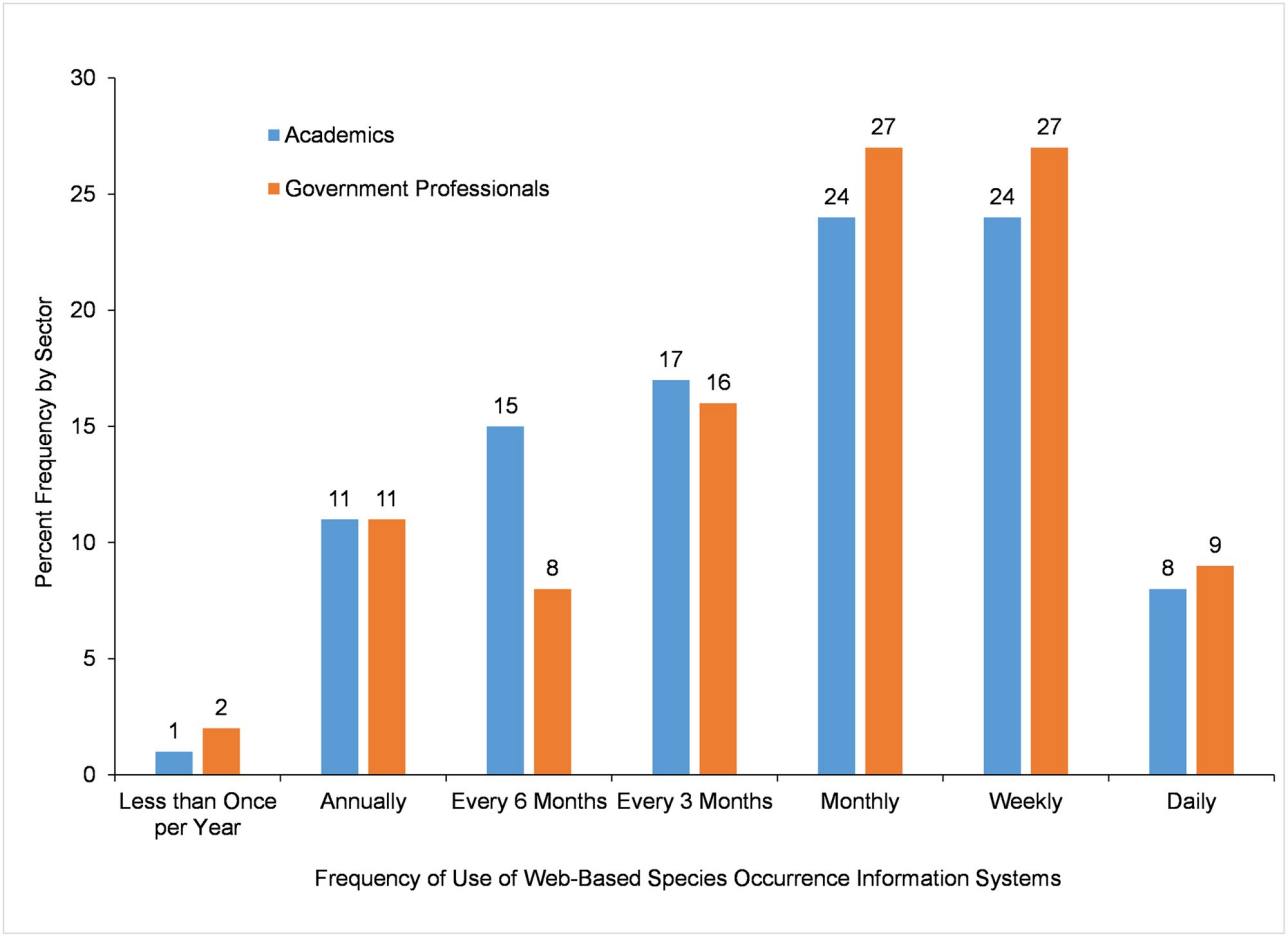
Frequency of use of web-based species occurrence information systems by participants who reported using those systems in the past 12 months.

The *Frequency of Use of Web-based Species Occurrence Information Systems* was reported by participants on a frequency scale ranging from *daily* to *less than once per year* (Fig 6). Results presented in Fig 6 focus only on participants who had used a web-based species occurrence information system in the last 12 months, and do not include participants who had not used those kinds of systems. There was no difference between academics and government professionals in mean ranks for *Frequency of Use of Web-based Species Occurrence Information Systems* (Table 11). Both *monthly* (24% academia vs. 27% government) and *weekly* (24% academia vs. 27% government) use of web-based species occurrence information systems were the most common use frequencies for both sectors of work.

**Table 11:** Mann-Whitney U tests of frequency of use of web-based species occurrence information systems.

Variable	U Statistic (Z)	P-value	Academia			Government		
			Number	Mean (SD)	Median (IQR)	Number	Mean (SD)	Median (IQR)
Frequency of Use of Web-based Species Occurrence Information Systems	54896.000 (-1.318)	0.188	245	4.6 (1.5)	5 (3)	476	4.7 (1.5)	5 (2)

On average, academics and government professionals did not differ in the number of *Hours per Week Use Web-based Species Occurrence Information Systems* (t = -1.096, df = 552, P = 0.274), even though these same academics used the web for work activities (*Hours per Week Use the Web for Work*) more hours per week than government professionals (t = 3.649, df = 552, P < 0.001). Although the distributions of both variables (*Hours per Week Use Web-based Species Occurrence Information Systems* and *Hours per Week Use the Web for Work*) by sector were positively skewed and not normally distributed, their variances were homogeneous, and neither log10 (hours used + 1) transformations nor analyses with Mann-Whitney U tests yielded results that were different from those obtained with the t-tests. Therefore, academics were similar to government professionals in the number of hours per week they use web-based species occurrence information systems, with academics using web-based species occurrence information systems an average of 3.38 hours per week (std. dev. = 4.96, n = 189) and using the web an average of 19.56 hours per week (std. dev. = 12.71, n = 189), while government professionals used web-based species occurrence information systems an average of 3.90 hours per week (std. dev. = 5.35, n = 365) and the web an average of 15.66 hours per week (std. dev. = 11.47, n = 365). The median number of hours per week academics and government participants use web-based species occurrence information systems is 2.00 hours per week (IQR _academia_ = 3, IQR _government_ = 4). Overall, use of web-based species occurrence information systems account for 17% of academics’ time and 25% of government professionals’ time per week using the web.

**Fig 7:**
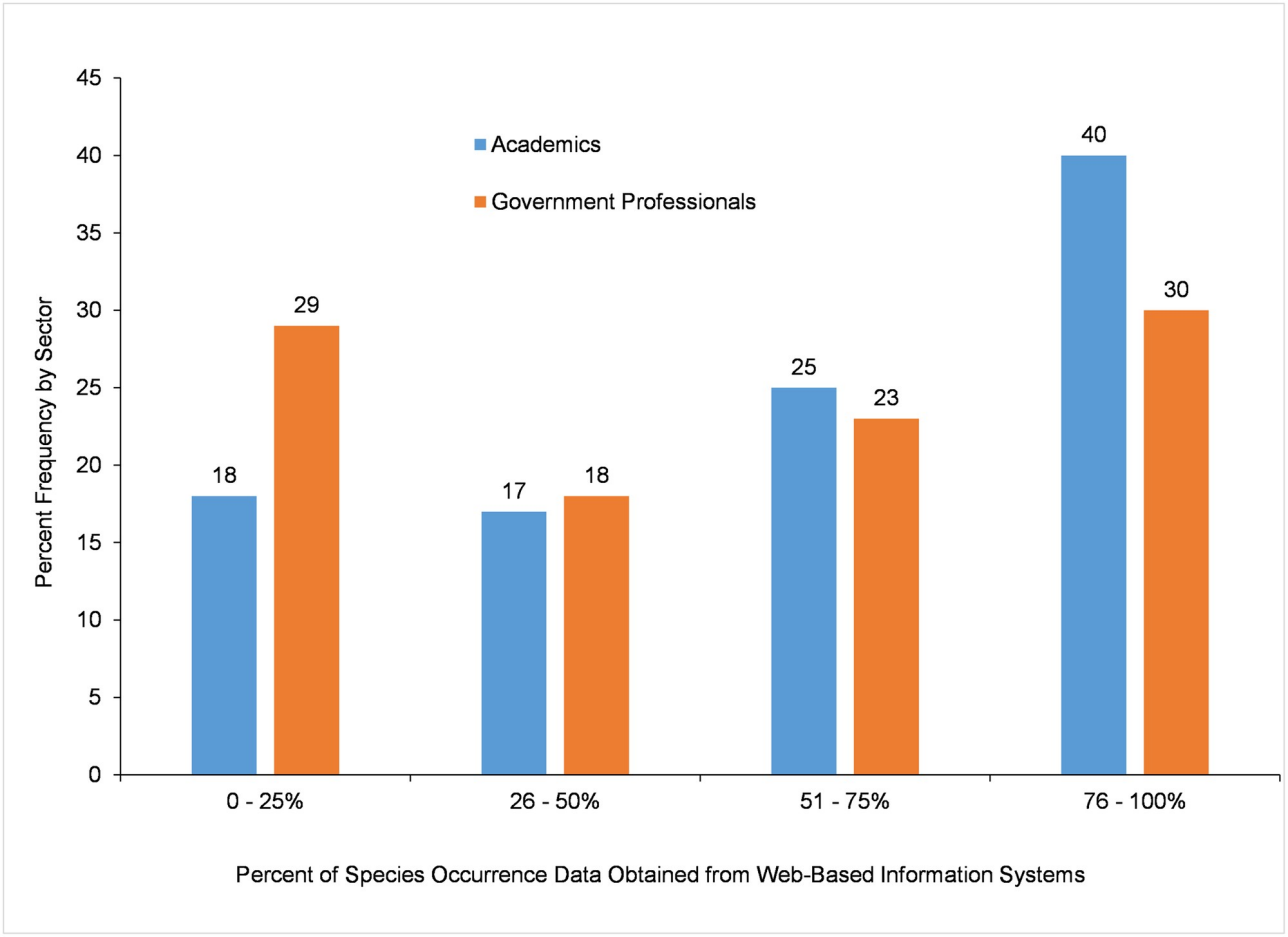
Percent of species occurrence data obtained by participants from web-based species occurrence information systems.

As shown in Fig 7, there were differences between sectors of work in the *Percent of Species Occurrence Data Obtained from Web-based Systems*. While 40% percent of academic participants (n = 244) reported that *76–100%* of their species occurrence data came from web-based species occurrence information systems, government participants (n = 473) were equally divided between those who obtained *76–100%* of all the species occurrence data they used from web-based species occurrence information systems and those for whom only a small percentage (*0–25%*) of all their data came from those systems (χ^2^ = 12.364, df = 2, P = 0.006, n = 717).

Participants were asked if, in addition to species occurrence data, they used other types of biodiversity data from web-based information resources. A higher percent of academic (65%, n = 238) than government (53%, n = 458) participants responded they use other biodiversity data from web-based information resources (χ^2^ = 9.318, df = 1, P = 0.002, n = 696).

### Perceptions about web-based species occurrence information systems

Participants who used species occurrence information systems were asked to rate the ease or difficulty they experienced with the following seven aspects of information system use and the data those systems provide: *Finding Systems*, *Accessing Systems*, *Using Systems*, *Identifying Data in Systems*, *Understanding Data Provided by Systems* (context of data), *Evaluating Quality of Data Provided by Systems* (data quality and trustworthiness), and *Retrieving Data in Needed Format*. Equal to or more than 69% of participants in each sector reported that *Finding Systems* and *Accessing Systems* was easy (responses included *somewhat easy*, *easy* and *extremely easy*), while 64% or more of participants in each sector indicated that *Using System*s was an easy aspect of information system use (Table 12).

**Table 12:** Level of ease / difficulty of aspects related to using web-based species occurrence information systems.

Variable	Category	Sector of Work	
		Academia % Frequency	Government % Frequency
Finding systems	Extremely difficult	0	0
	Difficult	3	2
	Somewhat difficult	12	12
	Neither easy nor difficult	15	16
	Somewhat easy	33	36
	Easy	30	28
	Extremely easy	8	5
	*Number participants (n)*	*(223)*	*(427)*
Accessing systems	Extremely difficult	1	0
	Difficult	2	2
	Somewhat difficult	8	12
	Neither easy nor difficult	18	17
	Somewhat easy	35	36
	Easy	28	28
	Extremely easy	8	5
	*Number participants (n)*	*(219)*	*(427)*
Using systems	Extremely difficult	1	0
	Difficult	1	2
	Somewhat difficult	14	9
	Neither easy nor difficult	19	22
	Somewhat easy	34	36
	Easy	24	27
	Extremely easy	6	4
	*Number participants (n)*	*(217)*	*(423)*

Category codes: extremely difficult (1), difficult (2), somewhat difficult (3), neither easy nor difficult (4), somewhat easy (5), easy (6), extremely easy (7).

In terms of aspects of a system related to the content (data) they provide, close to 50% of academic and government participants rated *Identifying Data in Systems* as overall easy (Table 13). At the opposite end of the scale, at least 50% of academic and government participants rated *Evaluating Quality of Data Provided by Systems* as the most difficult (responses included *somewhat difficult*, *difficult*, and *extremely difficult*) aspect of use of a web-based species occurrence information system (Table 13). *Understanding Data Provided by Systems* and *Retrieving Data in Needed Format* tended to be easy for some participants but difficult for others, and hence, these aspects of information system use were rated as somewhere in between easy and difficult overall.

**Table 13:** Level of ease / difficulty of aspects related to using data from web-based species occurrence information systems.

Variable	Level of Ease or Difficulty	Sector of Work	
		Academia % Frequency	Government % Frequency
Identifying data in systems	Extremely difficult	1	0
	Difficult	6	6
	Somewhat difficult	20	20
	Neither easy nor difficult	21	25
	Somewhat easy	25	29
	Easy	23	17
	Extremely easy	4	2
	*Number participants (n)*	*(217)*	*(417)*
Understanding data provided by systems	Extremely difficult	2	2
	Difficult	11	9
	Somewhat difficult	22	23
	Neither easy nor difficult	23	24
	Somewhat easy	26	27
	Easy	12	13
	Extremely easy	4	2
	*Number participants (n)*	*(218)*	*(430)*
Evaluating quality of data provided by systems **	Extremely difficult	9	5
	Difficult	24	18
	Somewhat difficult	30	27
	Neither easy nor difficult	18	24
	Somewhat easy	12	15
	Easy	5	9
	Extremely easy	2	2
	*Number participants (n)*	*(217)*	*(428)*
Retrieving data in needed format	Extremely difficult	3	1
	Difficult	8	10
	Somewhat difficult	22	28
	Neither easy nor difficult	24	21
	Somewhat easy	28	24
	Easy	13	13
	Extremely easy	2	2
	*Number participants (n)*	*(218)*	*(427)*

Category codes: extremely difficult (1), difficult (2), somewhat difficult (3), neither easy nor difficult (4), somewhat easy (5), easy (6), extremely easy (7).

** Statistically significant at P ≤ 0.01 (Mann-Whitney U test).

Mann-Whitney U test results (Table 14) indicate there are no statistically significant differences in the distributions and mean ranks of academics and government professionals for: *Finding Systems*, *Accessing Systems*, *Using Systems*, *Identifying Data in Systems*, *Understanding Data Provided by Systems*, and *Retrieving Data in Needed Format*. The only statistically significant difference in mean ranks between academics and government professionals is *Evaluating Quality of Data Provided by Systems* (Table 14), with academics tending to have more difficulty in evaluating quality and trustworthiness of data than government professionals.

**Table 14:** Mann-Whitney U tests of ease or difficulty with aspects of information system use.

Aspect of Information System Use	U Statistic (Z)	P-value	Academia			Government		
			Number	Mean (SD)	Median (IQR)	Number	Mean (SD)	Median (IQR)
Finding systems	45226.5 (-1.089)	0.276	223	5.0 (1.2)	5 (2)	427	4.9 (1.2)	5 (2)
Accessing systems	44810.5 (-0.901)	0.368	219	5.0 (1.2)	5 (2)	427	4.9 (1.2)	5 (2)
Using systems	45094.0 (-0.376)	0.707	217	4.8 (1.2)	5 (2)	423	4.9 (1.1)	5 (2)
Identifying data in systems	43188.0 (-0.964)	0.335	217	4.5 (1.4)	5 (3)	417	4.4 (1.2)	4 (2)
Understanding data provided by systems	46568.0 (-0.137)	0.891	218	4.1 (1.4)	4 (2)	430	4.1 (1.3)	4 (2)
Evaluating quality of data provided by systems **	39741.0 (-3.063)	0.002	217	3.3 (1.4)	3 (2)	428	3.6 (1.4)	3.5 (2)
Retrieving data in needed format	44228.0 (-1.060)	0.289	218	4.2 (1.3)	4 (2)	427	4.1 (1.3)	4 (2)

Category codes: *extremely difficult* (1), *difficult* (2), *somewhat difficult* (3), *neither easy nor difficult* (4), *somewhat easy* (5), *easy* (6), *extremely easy* (7).

** Statistically significant at P ≤ 0.01.

Participants were also asked to rate the *Importance of Web-based Species Occurrence Information Systems* for the work they do. The vast majority of academics (96%, n = 244) and government professionals (90%, n = 474) who used web-based species occurrence information systems rated the systems as important (responses included *somewhat important*, *important*, and *extremely important* categories). A Mann-Whitney U test (Table 15) indicated that importance of availability of these systems was higher for academics than for government professionals.

**Table 15:** Mann-Whitney U tests of importance, usefulness, and likelihood of using a web-based information system.

Variable	U Statistic (Z)	P-value	Academia			Government		
			Number	Mean (SD)	Median (IQR)	Number	Mean (SD)	Median (IQR)
Importance of availability information systems **	50275.5 (-3.032)	0.002	244	6.2 (1.0)	6 (1)	474	5.9 (1.2)	6 (2)
Usefulness of information systems used most **	48830.5 (-2.747)	0.006	237	6.2 (0.8)	6 (1)	467	6.0 (0.8)	6 (2)
Likelihood of using an information system in next 12 months	51920.0 (-1.936)	0.053	239	6.6 (0.8)	7 (1)	470	6.4 (1.0)	7 (1)

Category codes for Importance: *extremely unimportant* (1), *unimportant* (2), *somewhat unimportant* (3), *neither important nor unimportant* (4), *somewhat important* (5), *important* (6), *extremely important* (7).

Category codes for Usefulness: *extremely useless* (1), *useless* (2), *somewhat useless* (3), *neither useful nor useless* (4), *somewhat useful* (5), *useful* (6), *extremely useful* (7).

Category codes for Likelihood: *extremely unlikely* (1), *unlikely* (2), *somewhat unlikely* (3), *neither likely nor unlikely* (4), *somewhat likely* (5), *likely* (6), *extremely likely* (7).

** Statistically significant at P ≤ 0.01.

**Fig 8:**
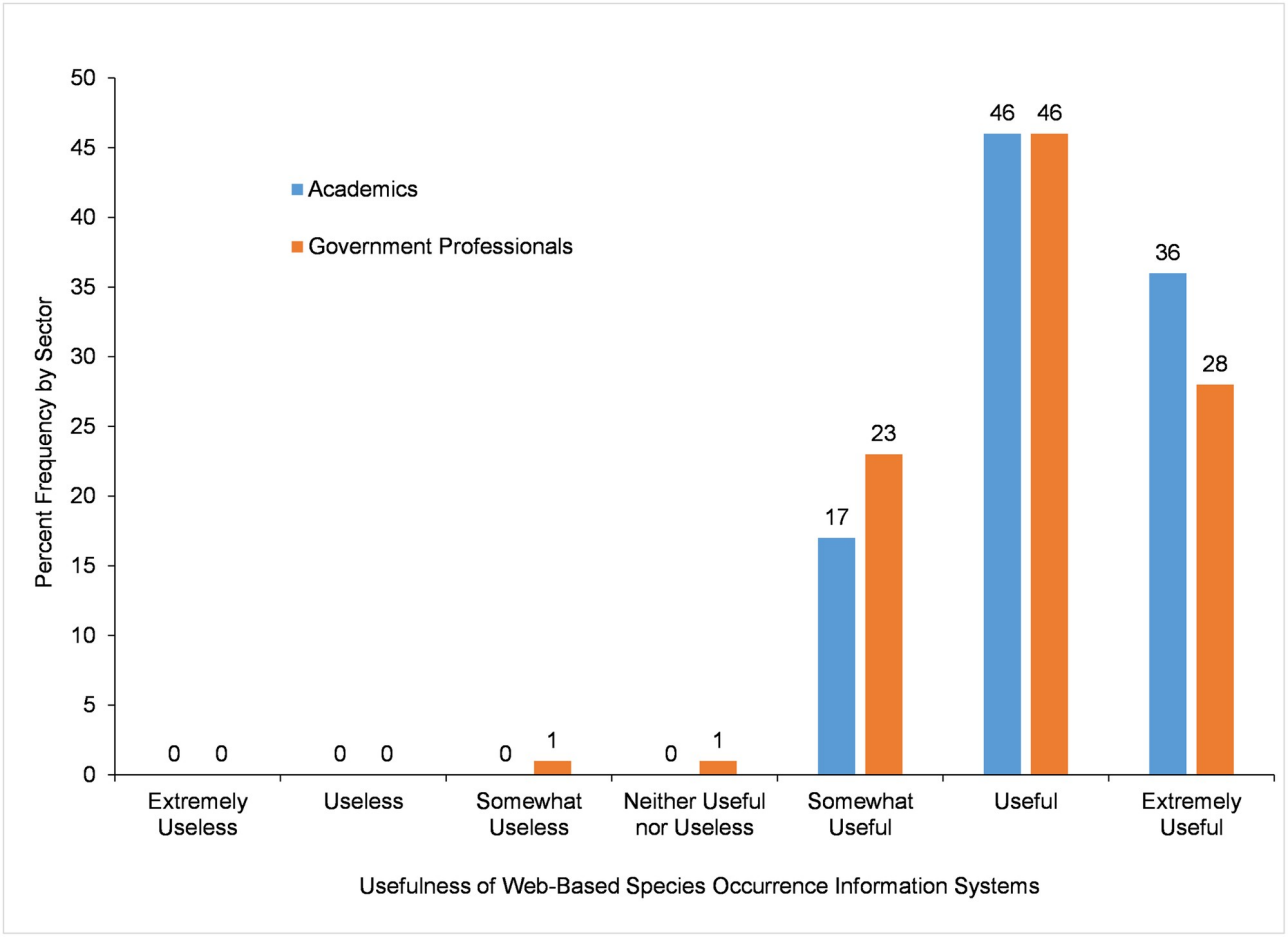
Level of usefulness of web-based species occurrence information systems.

Participants were also asked to rate the *Usefulness of Web-based Species Occurrence Information Systems* used the most. Almost all academic (99%, n = 237) and government (97%, n = 467) participants viewed the web-based species occurrence information systems they use most as useful (Fig 8) (responses included *somewhat useful*, *useful*, and *extremely useful*), with academics rating the usefulness of these systems higher than government professionals according to results of a Mann-Whitney U test (Table 15). The distribution of participant responses on *Usefulness of Web-based Species Occurrence Information Systems* was similar to that of participants’ responses on *Importance of Web-based Species Occurrence Information Systems*, and a moderately positive monotonic correlation was found between these variables (r_s_ = 0.516, n = 702, p<0.001) based on a Spearman’s correlation analysis.

### Awareness of web-based species occurrence information systems

Participants were asked about their level of awareness and/or use of nine national and global web-based species occurrence information systems (the number in parentheses is the year the system was launched online): Biodiversity Information Serving Our Nation (BISON) (2013) [87], Encyclopedia of Life (EOL) (2007) [88, 89], Global Biodiversity Information Facility (GBIF) (2001) [90], Integrated Digitized Biocollections Portal (iDigBio) (2013) [91], Map of Life (MOL) (2012) [92, 93], NatureServe Explorer (2000) [94, 95], Ocean Biogeographic Information Facility (OBIS) (2000) [96], PLANTS Database (1996) [97, 98], and VertNet (2013) [99, 100].

The systems *Used in last 12 months* by the highest percent of academic participants were *GBIF* (35%), then *EOL* (24%), the *PLANTS Database* (22%) and *iDigBio* (20%) (Table 16). The systems *Used in last 12 months* with the highest percent use by government participants were *NatureServe Explorer* and the *PLANTS Database*, both with 35% of participants using each system, followed distantly by *BISON* at 13%, *EOL* at 10%, and *GBIF* at 8% (Table 16). If we combine awareness categories *Used in last 12 months* and *Used long ago* as an overall indicator of use at some point in time, then the highest percentage of participants who used a system is about 50% for each sector of work, with 51% of academic participants using *GBIF* and 47% using *EOL*, while 52% of government participants use *NatureServe Explorer* and 43% use the *PLANTS Database*.

**Table 16:** Level of awareness of specific web-based species occurrence information systems.

Species Occurrence Information System	Level of Awareness	Sector of Work		Chi-square test of independence
		Academia % Frequency	Government % Frequency	
BISON	Never heard of	57	52	χ^2^ = 2.372
	Heard of but no used	26	27	df = 3
	Used long ago	7	8	P = 0.499
	Used in last 12 months	10	13	n = 699
	*Number participants (n)*	*(232)*	*(467)*	
EOL ***	Never heard of †	16	49	χ^2^ = 80.329
	Heard of but no used	37	30	df = 3
	Used long ago †	23	12	P < 0.001
	Used in last 12 months †	24	10	n = 697
	*Number participants (n)*	*(234)*	*(463)*	
GBIF ***	Never heard of †	28	64	χ^2^ = 118.454
	Heard of but no used	22	21	df = 3
	Used long ago †	16	7	P < 0.001
	Used in last 12 months †	35	8	n = 699
	*Number participants (n)*	*(232)*	*(467)*	
iDigBio ***	Never heard of †	42	84	χ^2^ = 148.950
	Heard of but no used †	27	12	df = 3
	Used long ago †	11	3	P < 0.001
	Used in last 12 months †	20	2	n = 696
	*Number participants (n)*	*(235)*	*(461)*	
MOL ***	Never heard of †	57	73	χ^2^ = 30.152
	Heard of but no used †	31	22	df = 3
	Used long ago	5	4	P < 0.001
	Used in last 12 months †	6	1	n = 690
	*Number participants (n)*	*(230)*	*(460)*	
NatureServe Explorer ***	Never heard of †	45	30	χ^2^ = 44.884
	Heard of but no used †	25	18	df = 3
	Used long ago	17	17	P < 0.001
	Used in last 12 months †	12	35	n = 699
	*Number participants (n)*	*(231)*	*(468)*	
OBIS *	Never heard of †	64	76	χ^2^ = 9.735
	Heard of but no used †	23	16	df = 3
	Used long ago	6	4	P = 0.021
	Used in last 12 months	7	4	n = 692
	*Number participants (n)*	*(230)*	*(462)*	
PLANTS **	Never heard of	48	44	χ^2^ = 14.594
	Heard of but no used †	21	13	df = 3
	Used long ago	9	8	P = 0.002
	Used in last 12 months †	22	35	n = 707
	*Number participants (n)*	*(238)*	*(469)*	
VertNet ***	Never heard of †	59	85	χ^2^ = 56.389
	Heard of but no used †	21	8	df = 3
	Used long ago †	7	2	P < 0.001
	Used in last 12 months †	13	5	n = 692
	*Number participants (n)*	*(233)*	*(459)*	

* Statistically significant at P ≤ 0.05 (Chi-square test).

** Statistically significant at P ≤ 0.01 (Chi-square test).

*** Statistically significant at P ≤ 0.001 (Chi-square test).

^†^ Statistically significant z-score with Bonferroni correction P ≤ 0.05 for multiple comparisons.

Table 16 shows that academic participants were most likely to *Never heard of OBIS* (64%), followed by *VertNet* (59%), and *MOL* and BISON both at 57%. Government participants were most likely to *Never heard of VertNet* (85%), *iDigBio* (84%), *OBIS* (76%), and *MOL* (73%). Except for *BISON*, awareness and/or use of the following information systems was dependent on sector of work: *EOL*, *GBIF*, *iDigBio*, *MOL*, *NatureServe Explorer*, *OBIS*, *PLANTS Database*, and *VertNet* (Table 16).

Based on results from z-scores of adjusted standardized residuals with a Bonferroni correction for each information system that had a statistically significant chi-square test, the greatest contribution to the significance of chi-square tests were from *Never heard of* and *Used in last 12 months*. The percentage of participants who selected *Never heard of* were significantly different between academics and government professionals for *EOL*, *GBIF*, *iDigBio*, *MOL*, *NatureServe Explorer*, *OBIS*, and *VertNet*. Only for the *PLANTS Database* and *BISON* were the percentage of participants who selected the category *Never heard of* similar between academics and government professionals. For awareness category *Used in last 12 months*, there were significant differences between academics and government professionals for *EOL*, *GBIF*, *iDigBio*, *MOL*, *NatureServe Explorer*, *PLANTS Database*, and *VertNet*. Only for *BISON* and *OBIS* were there similar percentages in *Used in last 12 months* for academics and government professionals. For *Heard of but not used*, there were differences in percent responses between academics and government professionals for *iDigBio*, *MOL*, *NatureServe Explorer*, *OBIS*, *PLANTS Database*, and *VertNet*. There were also significant differences between academics and government professionals for *Used long ago* for *EOL*, *GBIF*, *iDigBio*, and *VertNet*.

### Likelihood of continued use of web-based species occurrence information systems

**Fig 9:**
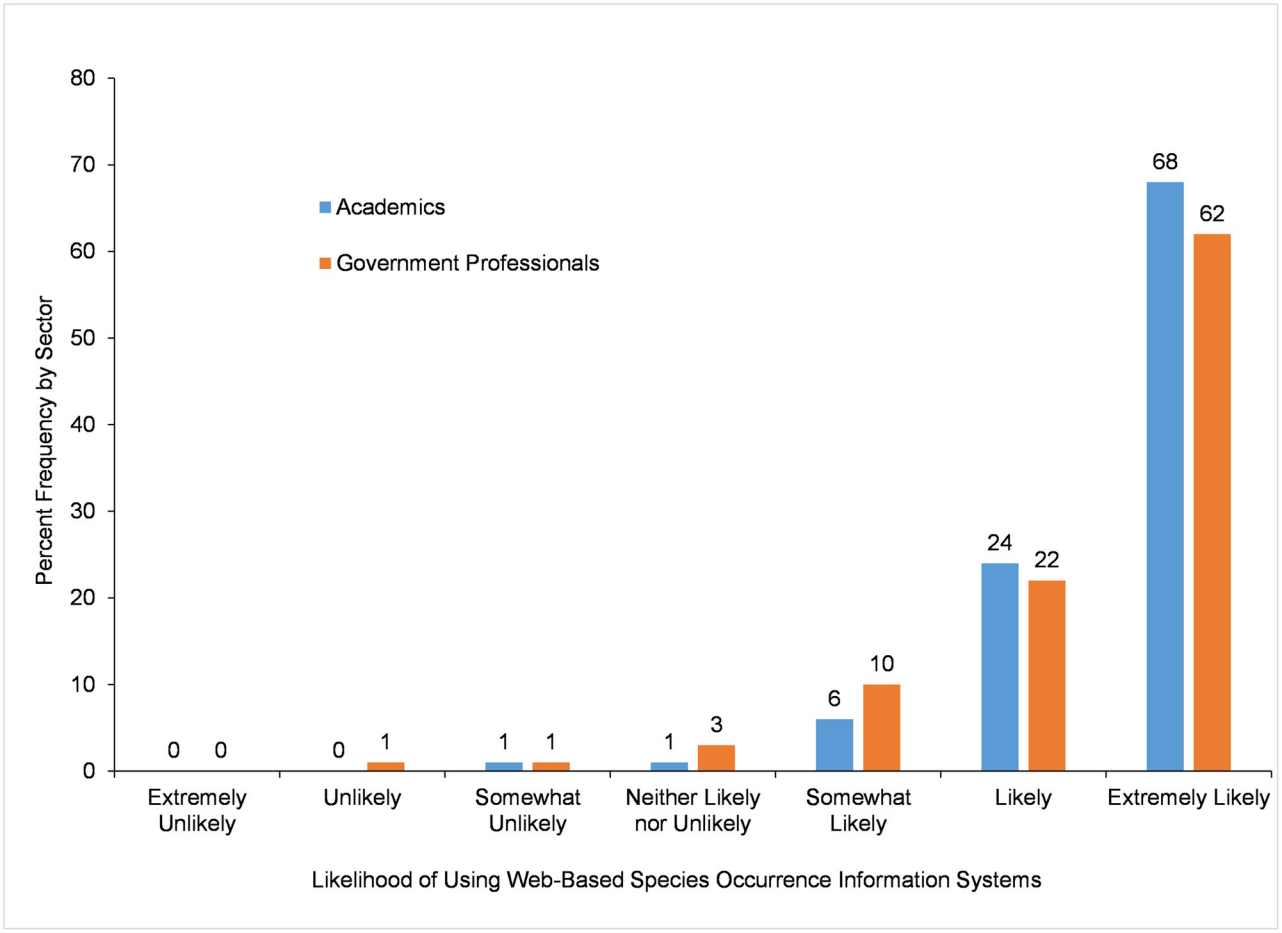
Likelihood of using a web-based species occurrence information system in the next 12 months.

As an indicator of intention to use an information system, participants were asked about their *Likelihood of Using Web-based Species Occurrence Information Systems* in the next 12 Months. Overall, 98% (n = 239) of academic and 94% (n = 470) of government participants reported they are likely (responses included *somewhat likely*, *likely*, and *extremely likely*) to use a web-based species occurrence information system in the next 12 months (Fig 9), with degree of likelihood not differing by sector according to results of a Mann-Whitney U test (Table 15).

Spearman’s correlation analyses were conducted to examine if there is a relationship between the participants reported *Likelihood of Using Web-based Species Occurrence Information Systems* and *Usefulness of Information Systems*. A moderate positive monotonic correlation was found between *Likelihood of Using Web-based Species Occurrence Information Systems* and *Usefulness of Information Systems* for both sectors combined (r_s_ = 0.530, n = 699, p<0.001), as well as for the academic sector (r_s_ = 0.503, n = 235, p<0.001) and government sector (r_s_ = 0.538, n = 463, p<0.001) separately.

## Discussion

### Limitations

The results of our study indicate that perceptions about web-based species occurrence information systems are similar between academics and government professionals in the United States, but the types of data they prefer and the systems they use appear to be different. Although our findings provide insights about areas for potential improvements in web-based species occurrence information systems, there are some potential limitations in our results due to the nature of online surveys, the statistical tests used to analyze data, and our sampling strategy to recruit participants for the study.

This study was based on a voluntary online survey of academics and government professionals who were invited to participate in the study. With voluntary survey instruments that are self-administered, there is the potential that non-response bias could occur if those who did not respond to our survey shared specific perspectives or experiences about web-based species occurrence information systems that were not shared by those who chose to participate in our survey. Bias could also have been introduced if those who participated in the survey overestimated or underestimated their average response to questions in the survey [101]. Although we did not control for non-response bias nor for biases that may have been introduced by the participants, by including participants who did not use web-based species occurrence information systems in our survey, we aimed for broader representation of perspectives about use of species occurrence data.

We used nonparametric tests (i.e. chi-square test, Mann-Whitney U test, and Spearman’s rank-order correlation) mostly to account for unequal sample sizes between sectors of work and skewed/non-normal distributions of variables consisting of nominal and ordinal data types. By relying on these tests, it is possible that significant differences between academics and government professionals on some variables may have been missed. Nonparametric tests tend to be less powerful than parametric tests in finding a significant difference when there is actually one, and in instances where violation of assumptions of parametric tests are not severe, parametric tests usually provide more accurate results [79]. Therefore, our conservative approach to analyzing data could have potentially limited our findings in this study.

Additional limitations of our study results may have resulted from sampling bias due to our methodology to recruit survey participants. Because we used websites of institutions and government agencies to look for names and emails of potential invitees to the survey, species occurrence data and information system users who were not listed in institutional websites were excluded from the potential pool of relevant participants for this study. We used this strategy however, because we felt that identifying potential invitees to our survey from institutional websites rather than from lists of academic peer-reviewed literature, one information system, or traditional scientific societies, would expand coverage to those who may not partake in those activities or be members of selective groups, since those are not traditional venues for recruiting government professionals. To limit the large number of institutions included in the CCIHE [67], which we used to identify universities for academic invitees to the survey, we only sampled doctoral universities with highest research activity, and therefore excluded participants from universities and colleges not focused on high research output. Nicholas et al. [26] found different use of electronic journals across universities differing in size and research activity. This suggests that data needs and use of data from web-based information systems could potentially be different for researchers at medium and small-sized universities where the primary focus is not research. New surveys of academics at universities and colleges where the focus is not research will be needed to determine if their use of species occurrence data and web-based species occurrence information systems is comparable to that of academics working at universities where research is the main focus.

There was a greater number of both academic and government participants from the South and West regions of the U.S. than from the other regions of the country. This suggests that although the two sectors of work were comparable in participating in the survey, there were geographic differences in participation within each sector. The method of sampling and recruiting academic participants from doctoral universities with highest research activity listed in the CCIHE [67] may have led to potential geographic bias in participation as some states may have not been as well represented in the CCIHE [67] list. For government professionals, although state-level organizations were identified for all states, only national and regional/sub-regional offices of federal agencies were included in our recruitment strategy, and therefore not all states were represented by federal government participants in this study. Because of potential geographic bias in results of this study, our findings may not be representative of professionals in all regions and states of the country, and certainly do not reflect the perspectives of professionals in countries other than the U.S.

In terms of demographics of our study participants, when compared to the broader academic and non-academic populations of biological science professionals in the United States [102], professionals between the ages of 18 to 34 years old were underrepresented in our study. The lower number of participants in that age group in our study may have been the result of our focus on academic faculty listings and government employees’ lists in websites. It is possible that young professionals may enter these organizations’ workforce via post-doctoral appointments or contract work, and hence why they were underrepresented in our study. Because of this, our findings may not represent the perspectives of the youngest generation of professionals that are entering the workforce, who tend to be tech savvy and probably are more at ease using web-based species occurrence information systems than older professionals. Additional studies will be needed to examine the species occurrence data and web-based species occurrence information system uses of young professionals.

Although non-academic participants were for the most part professionals who worked for government, there were a few participants who had indicated working for non-profit organizations or a for-profit business. Responses from those participants were grouped with those of government sector participants in our data analyses. The nature of work done by many non-profit organizations may be somewhat similar to the type of work done in government in relation to species management and conservation and, at least in the United States, non-profit organizations conduct work for government agencies via contracts and agreements. As these sub-sectors share some similarities, results from this study could potentially be applicable to professionals working in non-profit organizations. Additional studies of professionals in non-profit and for-profit sub-sectors will be needed to confirm what similarities may hold across non-academic sub-sectors of work.

### Users of species occurrence data and web-based species occurrence information systems

The gender distribution of academic and government participants, with a ratio of 2:3 females to males, is similar to what is found in the broad population of life sciences professionals in academia and the federal government [102]. Participant’s mean and median age category (41–50 years old) are also comparable to national statistics of employed scientists and engineers in the U.S. [102]. However, our sample includes relatively few participants from the ages of 18 to 34 years old for each work sector, and therefore the youngest professionals are underrepresented in our study. Except for the youngest professionals, the demographic characteristics of our study participants were comparable to the broader populations of life sciences professionals in academia and the federal government [102], and hence our results may be generalizable to life sciences professionals in the U.S. who work for those sectors and who use species occurrence data and web-based species occurrence information systems.

Academics held primarily doctorate degrees, while government professionals held either master’s degrees or to a lesser extent bachelor’s or doctorate degrees. While participants from academia worked primarily on scientific research in the biological sciences, government participants worked primarily on natural resource professions, specifically on species management and conservation, and land/water management and conservation. Perhaps due to the different type of work they do, academics and government professionals also differed in the geographic scope of the work they conduct, with academics tending to conduct work that is national and international in scope, while government professionals conduct work primarily at state and regional levels. Academic and government participants resided in similar regions of the country.

### Use of species occurrence data

Both academics and government professionals were similar in their self-reported level of experience with use of species occurrence data, and in using species occurrence data from others in their work. The two groups differed, however, in the preferred level of processing of the species occurrence data they use. Academics by far preferred original untransformed data, while government professionals showed preference for both original untransformed and summarized/synthesized data. The result that government professionals seem to have two preferred levels of data processing when obtaining data to use is not explained by the work they do, since the greatest percentages of those in government who prefer original untransformed data are working in natural resource professions rather than in scientific research. This result that government professionals prefer raw and summarized data supports the findings by Davis et al. [20] that government professionals in the Southeast United States have an equal need for raw and summarized biodiversity data. However, as in our study academics had a clear preference for raw over summarized data, our findings are not necessarily consistent with those of Davis et al. [20] that academics have an equal need for raw and summarized biodiversity data. It is possible that need for a data type may not as easily translate into preference for a data type. Alternatively, the focus of our study on use of data from others and use of only one type of biodiversity data, namely species occurrence data, may account for this difference in needed versus preferred data type for academics in our study.

Academics and government professionals use observational data and species ranges and distributions the most, with over 85% from each sector using those types of data. While academics use observational data and species ranges and distributions every 3 months, government professionals use these types of data on a monthly basis. The need for observational data by academic researchers has recently been documented across disciplines [103], and our results support those findings suggesting that observational data is the data type that is used the most in academia. Both academics and government professionals use instrument data at a similar frequency, every 6 months. The data types with the least frequency of use are citizen science data, especially for academics who tend to never or rarely use it, and specimen data, particularly for government professionals, who usually never or rarely use this type of data.

There may be various reasons why these sectors do not use these types of data in their work. Government professionals may not use specimen data perhaps due to a lack of exposure to natural history collections and specimens in their education and training, unavailability of specimen data or a presentation that does not meet their needs, or simply they do not have a need for this type of data. For citizen science data, some of the most successful citizen science programs have been conducted by non-profit organizations or government agencies to serve as outreach, educational and participatory engagement tools in addition to data collection [104]. Therefore, academics may be less exposed to these data. Alternatively, academics may decide not to use citizen science data due to data quality criticisms [105, 106], or simply because they have no need for this type of data in their work. Recognition of the potential citizen science data may have and how to correct data biases may not yet be realized in the broadest sense in the communities surveyed. New research is needed to explore the reasons why academics and government professionals use or do not use particular types of data.

### Sources of data and information

The most used sources of species occurrence data for academics and government professionals are colleagues and the web, with our definition of web including websites, search engines, web-based species occurrence information systems, and other web-based biodiversity resources. Both sectors use the web monthly to every 3 months, while colleagues are used as a source of data on average every 6 months by academics and every 3 months by government professionals, in addition to use of publications about every 6 months by academics and use of reports every 3 months by government professionals. Use of publications by academic scientists as a primary source of data has been well documented for various disciplines [24, 25, 27, 29, 30, 31, 34, 36, 66], and the use of colleagues as a primary source of biodiversity information has been reported for academic and non-academic professionals in the United States [20, 25]. With increased access to electronic data and information resources, scientists are increasingly using web resources to obtain data and information for use [25]. Findings by Hemminger et al. [25] that researchers were using web resources more than personal communications when searching for information, may extend to data searching as well, since in our study academic scientists used the web on par or even more than colleagues and publications as sources of species occurrence data. However, findings that publications were used more than any other information source by scientists [25, 28] do not appear to extend to data seeking strategies.

The slightly greater use of the web by academics in our study to obtain data for use when compared to their use of publications, might be a result of our narrower focus on species occurrence data when compared to previous studies that focused on seeking practices for broader types of information. Species occurrence data are collected and/or generated in both academic and non-academic settings, and therefore, can be found in sources outside of publications. Some of the greatest volume of species occurrence data are observational data gathered by government programs and citizen science programs for certain taxonomic groups of species [107, 108], where volume, duration, and use of the data collected likely surpasses those generated or provided via academic publications. Therefore, the volume of data, duration of data collection efforts, and the dissemination mechanism for certain data types may impact the frequency by which a data source is used by particular communities of professionals. Alternatively, greater use of the web in our study by academics when compared with previous studies of academic scientists may also be due to our broad definition of the web in our questionnaire. Our definition of web included not only web-based species occurrence information systems but also search engines and other websites. The use of search engines [28, 45, 109] and other websites [50] by scientists to access data and information, including publications, is well documented. It is very likely that use of the web by our study participants meant they were using web resources such as search engines, websites, and web-based information systems to find species occurrence data for use. Our study found that the web is also considered an important source of data for government professionals. These results on use of the web by academics and government professionals suggest that now that web resources have been around for a longer period of time and are more ubiquitous, both of these sectors are using the web as a primary source of data on par with their other preferred data sources.

Of those participants who used web-based species occurrence information systems, both academics and government professionals learned about web-based species occurrence information systems from colleagues, and none of the other sources seemed as effective in conveying information about the existence of these systems to potential users. After colleagues, web search engines were a very distant second source used by participants to learn about the existence of web-based species occurrence information systems. It appears that use of web search engines by academics to find data or data repositories yields mixed results [40]. Therefore, information system developers should not rely on web search engines as an only means to make their information systems discoverable. This has implications for design of effective information and data dissemination approaches for web-based species occurrence information systems. Given how colleagues figure prominently as sources of information for web-based species occurrence information systems, the human dimensions and social networks (not just electronic but human networks in general) of professionals should figure more prominently in outreach and communication strategies aimed at improving discoverability of web-based species occurrence information systems. A review of data retrieval practices across scientific disciplines found that data retrieval is a process that involves not only the user and the information system, but rather includes the user’s social networks and personal exchanges as the user navigates finding, obtaining and evaluating data for his or her use [45]. Our results support this broader view of data retrieval as a process that involves social aspects when using data from web-based species occurrence information systems. There is recognition that as new information discovery methods emerge and the information landscape becomes more diverse, new collaborations and approaches are needed to increase discoverability of scholarly information resources [110]. Therefore, research should be undertaken to identify what approaches work best in disseminating information about available systems and data via these human/social networks of professionals.

### Use of web-based information systems

On average, both academics and government professionals use web-based species occurrence information systems on a monthly basis, and they tend to use them for about 2–4 hours per week. A greater percentage of the data used by academics came from web-based information systems when compared to government professionals, with more than 50% of the species occurrence data used by over half of academic participants coming from those systems. Academics also use more than government professionals other types of biodiversity data from web-based information resources.

### Perceptions about web-based species occurrence information systems

Perceptions about various aspects of information system use appear to be similar for academics and government professionals. The aspects most participants found easy when using species occurrence information systems were finding systems, accessing a system, and using a system. The aspect that participants found most difficult when using information systems was evaluating quality of data provided by a system, especially for academics. These results show that, both academics and government professionals tend to have more difficulty with aspects related to the data (content) provided by web-based species occurrence information systems rather than with the technology or electronic systems themselves.

Our finding that academics had more difficulty with data quality evaluation than government professionals was unexpected. In disciplines like biomedicine, data users from different specialties use different proxies for assessing data quality, and definitions of what constituted quality in data varied by the user [40]. Therefore, it is possible that what defines data quality and how it is assessed may be different for the two sectors in our study, and that academics may need more information and details to assess quality of data than government professionals.

Studies of academic researchers on how they develop trust in data indicate that users utilize several of the following lines of evidence to assess data trustworthiness: competence of the data producer, expert recommendations from colleagues (including peer review), own experience with data, intrinsic quality of the data, data documentation and management, and data repository (intermediary) reputation [51, 111, 112]. Some of these lines of evidence are not available or easily accessed from web-based species occurrence information systems, and this may contribute to the difficulty experienced by academics when evaluating the quality of data from these systems. Furthermore, data evaluation needs may be different for academics and government professionals given our findings about their preferences for different web-based information systems and types of species occurrence data (raw versus summarized data).

Increased emphasis should be placed on research and activities that seek to understand how to better convey quality, context, social aspects (e.g. competence, reputation) and intricacies of the data that are provided by these systems to users. Although the biodiversity informatics community has made progress in providing metadata and visualizations about data in many of these information systems, it appears these efforts are not sufficient in helping users understand, evaluate, and use those data. Gregory et al. [45] provide a conceptual framework that views data search as a socio-technical practice. In that framework, the data seeking process includes aspects that are social, such as relying on colleagues and other people’s expertise to find data and develop trust in the data producers, and aspects that are technical, such as retrieving data from systems and conducting exploratory analyses to determine if data fall within acceptable ranges. Therefore, not only is there a need for improving quality of the data provided by web-based species occurrence information systems, but also for development and implementation of methods that help convey the social aspects of data search and use, as well as metrics that help assess the level of content understanding by users to be able to truly evaluate the effectiveness of these systems in meeting the needs of users.

### Awareness of web-based species occurrence information systems

Even though participants in this study indicated that on average finding information systems that provide species occurrence data was easy, a large percentage of participants were not aware of the existence of many of the national and global web-based species occurrence information systems listed in the survey. Moreover, academics and government professionals tend to use different systems and differed in their awareness about the existence of specific systems. Systems that were usually known to academics were the systems unknown to government professionals and vice versa. The most used systems overall by academics were GBIF and EOL, while for government professionals were NatureServe Explorer and the PLANTS Database. Based on our results, it appears that current outreach activities conducted for most species occurrence information systems have failed to reach a large proportion of these professionals. Although our study did not collect data on why some of these web-based species occurrence information systems were known or used more than others, our results may suggest areas of potential focus. For example, when comparing percent frequencies of use of each system by sector, academics appeared to use most those systems originally developed by academic communities (GBIF) or natural history museums (EOL), while government professionals appeared to use more those systems developed by non-profit organizations (NatureServe Explorer) or government agencies (PLANTS Database). Our findings indicate that professionals learned about the existence of information systems primarily from colleagues. Since information from colleagues conveys not only the existence of a resource, but often aspects related to evaluation and trust in the information system from the user’s own community of practice, it is likely that they would gravitate towards information systems that are developed within their own professional communities, since data and information seeking appear to be social processes. Additional research is needed to examine whether information seeking behavior of academic and government professionals, system longevity, or certain preferred system characteristics may have contributed to greater awareness and use of some of these systems by professionals who use species occurrence data.

### Likelihood of continued use of web-based species occurrence information systems

In our study, the vast majority of academics and government professionals who used web-based species occurrence information systems indicated they were likely to use one of these systems within a 1-year timeframe. Intention to use an information system has often been considered as a predictor of use of closed information systems and may serve as a proxy for examining adoption and continued use of open-access information systems depending on the type of predictor used [10, 60]. The most widely used models for acceptance of an information system incorporate intention to use an information system, perceived usefulness, and perceived ease of use as predictors of actual use of a system [10, 61, 62, 63]. In some studies, usefulness of an information system has been found to be correlated to usage, and perceived usefulness and ease of use appear to influence user satisfaction [58]. Given that in our study the majority of participants, especially academics, indicated web-based species occurrence information systems were important and useful to them, had a moderate to high likelihood of using an information system within a year, and the degree of usefulness was positively related to their likelihood of using an information system, it appears on first inspection that participants who used web-based species occurrence information systems in their work planned to continue using those systems in the future. Because perceptions such as perceived usefulness and other indicators of satisfaction are included in predictive models of information system use, understanding user behavior, attitudes, and perceptions are important for providing information resources that can meet users’ expectation. Additional research on these topics and a closer examination of the relationships and potential determinants of information system use will need to be conducted to better understand system use continuance and to clearly identify variables that may be actionable when it comes to developing and improving web-based species occurrence information systems to meet the needs of existing and potential new users.

## Conclusion

Academics working primarily in scientific research at institutions of high research activity and government professionals working on natural resources at government agencies tend to have similar views, perceptions, and preferences in terms of ease of use of various system aspects, the importance and usefulness of these systems for the work they do, the types of data they are looking for, and the sources they like to use to obtain species occurrence data and learn about new information systems. But even though these sectors are similar in many respects, they differ in significant ways with regards to the level of processing they prefer the data to be presented or provided to them, how much of the data they use come from information systems, and what types of systems they prefer to use. Therefore, when assessing how usable a system is and delivering species occurrence data via web-based species occurrence information systems to academics and government professionals, information system developers should take into consideration the sectors’ differences in preferred level of data processing and how they obtain data, and consider providing different views of their species occurrence data to each sector based on identified preferences. On first inspection, the content (data) aspects of information system use emerge from the perspective of users as the most difficult elements in their current use of web-based species occurrence information systems. It would therefore be useful to apply a socio-technical framework [45] to examine how discovery and use of species occurrence data from web-based information systems could be facilitated for users given their differences in data preferences and the data use processes followed by different user communities. By taking into account the preferences of user communities and the social and technical aspects of data search and use, information system developers in biodiversity informatics can help meet the data needs of user communities when planning for development or improvements to web-based species occurrence information systems.

## Supporting information

S1 Appendix Questionnaire of online survey. (PDF)

S2 Appendix Disciplines / Fields and type of agencies searched for potential invitees to the survey. (PDF)

S3 Appendix Disposition codes of online survey and relevant equations. (PDF)
