# Supplementary material for: Use of web-based species occurrence information systems by academics and government professionals
Source: PLoS One. 2020 Jul 31;15(7):e0236556. doi: 10.1371/journal.pone.0236556 (PMC7394390; doi:10.1371/journal.pone.0236556)
Supplement: S1 Appendix — (PDF) [file pone.0236556.s001.pdf]

S1 Appendix. Questionnaire of online survey.

[Note: Text in brackets denotes question number, logic used in the survey to show a question based on a previous answer, or numeric codes used for data summaries and analyses. This text was not shown to users when the survey was administered online.]

Informed consent

USE OF SPECIES OCCURRENCE INFORMATION SYSTEMS AND DATA REUSE BY NATURAL RESOURCE PROFESSIONALS AND RESEARCH SCIENTISTS

School of Natural Resources and Environment

University of Florida

IRB protocol # 2014-U-0321

This study will investigate the use of species occurrence data and the use of Web-based species occurrence information systems by natural resource professionals and research scientists. Information from this study will help increase our understanding of how species occurrence data are reused, and may also contribute towards enhancing species occurrence information systems to meet the needs of users.

Definitions: Species occurrence data are data about the occurrence of a species (or another taxonomic group) at a geographic location and point or period in time. Web-

based species occurrence information systems are databases and their data retrieval systems (or applications) provided via websites and the Internet to discover, access, visualize, summarize or download species occurrence data.

You will be asked to complete an online survey about your work-related use of species occurrence data that were generated or collected by someone other than you (your reuse of data from others), and your use (if any) of Web-based species occurrence information systems. As part of the survey, you will also be asked a few demographic questions since other research has shown that some demographic factors may influence use of information systems. By completing this survey, you are giving consent for the Principal Investigator to use your responses in this study.

The survey will take approximately 10 to 20 minutes to complete depending on whether or not you have used Web-based species occurrence information systems in the last year.

There are no anticipated risks, no direct benefits and no compensation for participating in this study. Your participation is completely voluntary and you can quit at any time.

The information collected in this online survey will be kept confidential to the extent provided by law. Your responses to the survey questions will not be associated with your name or any other identifying information when data is downloaded for analysis,

and will therefore be anonymous. All information collected will remain confidential and results will be reported only as aggregated data.

There is a minimal risk that security of any online data may be breached, but our survey host (University of Florida QUALTRICS) uses strong encryption and other data security methods to protect your information. Only the researchers will have access to your information on the Qualtrics server. If you have any questions or concerns about this study you may contact Elizabeth Martín (Principal Investigator and UF doctoral student) at [email], or Dr. Larry Page of the Florida Museum of Natural History at [email]. For questions regarding your rights as a research participant in this study you may contact the Institutional Review Board (IRB-02) Office, Box 112250, University of Florida, Gainesville, FL 32611-2250; telephone (352) 392-0433.
By selecting "Yes, I agree to participate in this survey" below, I acknowledge that I have read the information provided above, I am at least 18 years of age, and I voluntarily agree to participate in this survey.

☐ Yes, I agree to participate in this survey [1]

☐ I decline to participate in this survey [2]

Background and demographic Information

The first set of questions focuses on your work-related background. These questions may help us identify factors related to the reuse of species occurrence data and the use of Web-based information systems.

[Q2.3] What is your primary area of work?

Please select the area in which you spend the greatest amount of time.

(Please answer this question. Your answer will determine the questions that are shown next.)

☐ Scientific research [1]

☐ Teaching and education [2]

☐ Natural resource management, conservation and restoration practice [3]

☐ Environmental planning & review, land use planning [4]

☐ Policy, law and administration [5]

☐ Technical/scientific information transfer and communications [6]

☐ Other. Please specify.[90] ______________________________

[Q2.4] Please select the discipline that best represents the work you do, and specify the field.

[Display This Question: If 'Scientific research' is selected for Q2.3]

- ☐ Biological Sciences (e.g. Botany, Ecology, Evolution, Physiology, Zoology) [1] ______________________________
- ☐ Physical Sciences (e.g. Geography, Geology, Oceanography) [2] ______________________________
- ☐ Multidisciplinary or Applied Sciences (e.g. Agriculture, Fisheries, Forestry, Medicine) [3] ______________________________
- ☐ Mathematics, Statistics [4] ______________________________
- ☐ Computational Science, Informatics [5] ______________________________
- ☐ Planning, Architecture [6] ______________________________
- ☐ Other. Please specify the discipline and field. [90] ____________________________

[Q2.5] Please select the category that best represents the work you do.

(If you work on more than one of following categories, please select the category in which you spend the greatest amount of time.)

[Display This Question: If 'Natural resource management, conservation and restoration practice' is selected for Q2.3]

☐ Land/water management and conservation (e.g. site/area management, habitat and ecosystem restoration) [1]

☐ Species management and conservation (e.g. game management, species of concern assessment/recovery/reintroduction) [2]

☐ Nuisance species / invasive species control and management [3]

☐ Other. Please specify. [90] ________________________________

[Q2.6] Please select the category that best represents the work you do.(If you work on more than one of the following categories, please select the category in which you spend the greatest amount of time.)

[Display This Question: If 'Environmental planning & review, land use planning' is selected for Q2.3]

☐ Environmental planning [1]

☐ Environmental review [2]

☐ Land use planning [3]

☐ Other. Please specify. [90] ________________________________

[Q2.7] Please select the category that best represents the level at which you teach/educate.

[Display This Question: If 'Teaching and education' is selected for Q2.3]

☐ Post-secondary education (e.g. colleges, universities) [1]

☐ Primary or secondary education [2]

☐ Informal education [3]

☐ Other. Please specify. [90] ______________________________

[Q2.8] Please select the category that best represents the discipline in which you teach/educate at a post-secondary institution, and specify the field.

[Display This Question: If 'Post-secondary education (e.g. colleges, universities)' is selected for Q2.7]

☐ Biological Sciences (e.g. Botany, Ecology, Evolutionary Biology, Physiology, Zoology) [1] ______________________________

☐ Physical Sciences (e.g. Geography, Geology, Oceanography) [2] ______________________________

☐ Multidisciplinary or Applied Sciences (e.g. Agricultural Science, Fisheries, Forestry, Medicine) [3] ______________________________

☐ Mathematics, Statistics [4] ______________________________

☐ Computational Science, Informatics [5] ______________________________

☐ Planning, Architecture [6] ______________________________

☐ Other. Please specify the discipline and field. [90] ________________________

[Q2.9] In addition to teaching/education, do you also conduct scientific research in the discipline you selected for the previous question?

[Display This Question: If 'Post-secondary education (e.g. colleges, universities)' is selected for Q2.7]

☐ Yes [2]

☐ No [1]

[Q2.10] Please specify the field or sub-area in which you work.

[Display This Question: If 'Policy, law and administration' OR 'Technical/scientific information transfer and communications' is selected for Q2.3]

____

[Q2.11] If you want to provide additional information to help us better understand what your role is at work, you can provide that information here.

____

[Q2.12] Which of the following best describe the geographic scope of the primary work that you do? Please select all that apply.

☐ International (outside the U.S.) [1]

☐ National (U.S.) [1]

☐ Regional (U.S.) [1]

☐ State (U.S.) [1]

☐ Sub-region within a state (U.S.) [1]

☐ Local (U.S.) [1]

☐ Other not listed above. Please specify. [1] ______________________________

[Q2.13] Is your most current employment based in the United States or U.S. Territories?

☐ Yes [2]

☐ No. Please indicate in which country you are based. [1] ____________________

☐ Other. Please specify. [90] ______________________________

[Q2.14] In which state do you currently reside?

☐ Alabama [1]

☐ Alaska [2]

☐ Arizona [3]

☐ Arkansas [4]

☐ California [5]

☐ Colorado [6]

- ☐ Connecticut [7]
- ☐ Delaware [8]
- ☐ District of Columbia [9]
- ☐ Florida [10]
- ☐ Georgia [11]
- ☐ Hawaii [12]
- ☐ Idaho [13]
- ☐ Illinois [14]
- ☐ Indiana [15]
- ☐ Iowa [16]
- ☐ Kansas [17]
- ☐ Kentucky [18]
- ☐ Louisiana [19]
- ☐ Maine [20]
- ☐ Maryland [21]
- ☐ Massachusetts [22]
- ☐ Michigan [23]
- ☐ Minnesota [24]

☐ Mississippi [25]

☐ Missouri [26]

☐ Montana [27]

☐ Nebraska [28]

☐ Nevada [29]

☐ New Hampshire [30]

☐ New Jersey [31]

☐ New Mexico [32]

☐ New York [33]

☐ North Carolina [34]

☐ North Dakota [35]

☐ Ohio [36]

☐ Oklahoma [37]

☐ Oregon [38]

☐ Pennsylvania [39]

☐ Rhode Island [40]

☐ South Carolina [41]

☐ South Dakota [42]

☐ Tennessee [43]

☐ Texas [44]

☐ Utah [45]

☐ Vermont [46]

☐ Virginia [47]

☐ Washington [48]

☐ West Virginia [49]

☐ Wisconsin [50]

☐ Wyoming [51]

☐ U.S. Territories [52]

☐ I do not reside in the United States or U.S. Territories [999]

[Q2.15] In which sector do you currently work?

☐ Academia (colleges, universities) [1]

☐ State or local government [2]

☐ Federal government [3]

☐ Non-profit organization [4]

☐ For-profit business [5]

☐ Not currently working (e.g. retired, unemployed, student) [999]

☐ Other. Please specify. [90] ______________________________

The next four questions ask demographic information that may relate to the use of Web-based species occurrence information systems.

[Q2.17] What is the highest degree or level of education you have completed?

☐ Doctorate degree [7]

☐ Master's degree [6]

☐ Bachelor's degree [5]

☐ Associate degree [4]

☐ Some college, no degree [3]

☐ Trade / technical / vocational training [2]

☐ High school graduate (diploma or equivalent) [1]

☐ Other not listed above. Please specify. [90] ______________________________

[Q2.18] When did you receive your last degree or completed your highest level of education?

☐ Prior to 1960 [7]

☐ 1961 - 1970 [6]

☐ 1971 - 1980 [5]

☐ 1981 - 1990 [4]

☐ 1991 - 2000 [3]

☐ 2001 - 2010 [2]

☐ 2011 or later [1]

[Q2.19] How old were you on your last birthday?

☐ 81 years or more [7]

☐ 71 - 80 years [6]

☐ 61 - 70 years [5]

☐ 51 - 60 years [4]

☐ 41 - 50 years [3]

☐ 31 - 40 years [2]

☐ 18 - 30 years [1]

[Q2.20] What is your gender?

☐ Male [2]

☐ Female [1]

☐ Other [90]

[Q2.21] How many hours per week would you say on average that you use the Web for work?

____

Species occurrence data

The next questions focus on general use of species occurrence data. For these questions, the word 'use' includes using your own data as well as data from others in any aspect of your work. Species occurrence data are defined as data about the occurrence of a species (or another taxonomic group) at a geographic location and point or period in time. Species occurrence data are one type of biodiversity data. Examples of species occurrence data include species presence or absence, species distributions, species ranges.

[Q3.3] Which of the following best describes your use of species occurrence data?

(Please answer this question. Your answer will determine the questions that are shown next.)

☐ I use species occurrence data in my current position. [3]

☐ I do not currently use species occurrence data, but I have used it in the past. [2]

☐ I have never used species occurrence data. [1]

[Q3.4] Please rate your level of experience using species occurrence data.

[Display This Question: If ‘I use species occurrence data in my current position’ OR ‘I do not currently use species occurrence data, but I have used it in the past’ is selected for Q3.3]

☐ Very experienced [7]

☐ Experienced [6]

☐ Somewhat experienced [5]

☐ Neither experienced nor inexperienced [4]

☐ Somewhat inexperienced [3]

☐ Inexperienced [2]

☐ Very inexperienced [1]

☐ No opinion or undecided [999]

No use species occurrence data

[Q4.1] What are the primary reasons that you do not use species occurrence data in your work?

[Display This Question: If ‘I have never used species occurrence data’ is selected for Q3.3]

____

Data reuse

The next questions focus on reuse of species occurrence data from others. From this point forward in the survey, questions on species occurrence data will be about reuse of these data from others in your work. Data reuse means the use of data that were originally generated or collected by someone other than you and for a project/program different from yours.

[Q5.3] Do you reuse species occurrence data from others in your work?

(Please answer this question. Your answer will determine the questions that are shown next.)

[Display This Question: If ‘I use species occurrence data in my current position’ OR ‘I do not currently use species occurrence data, but I have used it in the past’ is selected for Q3.3]

☐ Yes [2]

☐ No [1]

No reuse species occurrence data

[Q6.1] What are the primary reasons that you do not reuse species occurrence data in your work?

[Display This Question: If ‘No’ is selected for Q5.3]

____

Your data reuse

[Q7.1] When acquiring species occurrence data for reuse, what kind of data do you prefer? *[Display This Question: If ‘Yes’ is selected for Q5.3]*

☐ Analyzed, modeled data (species ranges and distribution models & maps) [4]

☐ Summarized, synthesized data (graphs, summary tables, point occurrence maps) [3]

☐ Transformed, normalized data [2]

☐ Original, untransformed data (raw data) [1]

☐ Other. Please specify. [90] ____

☐ No preference [999]

[Q7.2] How often do you reuse the following types of species occurrence data?

[Display This Question: If ‘Yes’ is selected for Q5.3]

	Daily [7]	Weekly [6]	Monthly [5]	Every 3 months [4]	Every 6 months [3]	Annually [2]	Never or less than once per year [1]
Observational data from inventories, surveys, and monitoring programs [Q7.2_1]	☐	☐	☐	☐	☐	☐	☐
Data from specimens in natural history collections [Q7.2_2]	☐	☐	☐	☐	☐	☐	☐
Data from images, recordings, and instruments (e.g. remote sensing, DNA sequencing) [Q7.2_3]	☐	☐	☐	☐	☐	☐	☐
Data from citizen science programs [Q7.2_4]	☐	☐	☐	☐	☐	☐	☐
Species geographic ranges and distributions [Q7.2_5]	☐	☐	☐	☐	☐	☐	☐

[Q7.3] How often do you use the following data sources to obtain species occurrence data for reuse?

[Display This Question: If ‘Yes’ is selected for Q5.3]

	Daily [7]	Weekly [6]	Monthly [5]	Every 3 months [4]	Every 6 months [3]	Annually [2]	Never or less than once per year [1]
Colleagues / personal contacts [Q7.3_1]	☐	☐	☐	☐	☐	☐	☐
Organization / agency reports, assessments and planning documents [Q7.3_2]	☐	☐	☐	☐	☐	☐	☐
Books [Q7.3_3]	☐	☐	☐	☐	☐	☐	☐
Articles in trade publications and peer-reviewed journals [Q7.3_4]	☐	☐	☐	☐	☐	☐	☐
Web-based information resources and systems [Q7.3_5]	☐	☐	☐	☐	☐	☐	☐

Web-based species occurrence information systems

The next questions focus on awareness and use of Web-based species occurrence information systems.

The following definition applies: Web-based species occurrence information systems are information resources including databases and their data retrieval systems (or applications) provided via websites and the Internet to discover, access, visualize, summarize or download species occurrence data. These information resources are dedicated to providing species data and information.

[Q8.3] Have you used a Web-based species occurrence information system at least once in the past 12 months?

(Please answer this question. Your answer will determine the questions that are shown next.)

[Display This Question: If 'I use species occurrence data in my current position' OR 'I do not currently use species occurrence data, but I have used it in the past' is selected for Q3.3]

☐ Yes [2]

☐ No [1]

Non-use species occurrence information system in last 12 months

[Q9.1] Are you aware of colleagues in your line of work using Web-based species occurrence information systems?

[Display This Question: If ‘No’ is selected for Q8.3]

☐ Yes [2]

☐ No [1]

☐ Not sure [3]

[Q9.2] Have you heard of any of the following Web-based species occurrence information systems? Please select all that apply.

[Display This Question: If ‘No’ is selected for Q8.3]

☐ Biodiversity Information Serving Our Nation (BISON) [1]

☐ Encyclopedia of Life (EOL) [1]

☐ Global Biodiversity Information Facility (GBIF) [1]

☐ Integrated Digitized Biocollections (iDigBio) Portal [1]

☐ Map of Life (MOL) [1]

☐ NatureServe Explorer [1]

☐ Ocean Biogeographic Information System (OBIS) [1]

☐ PLANTS Database [1]

☐ VertNet [1]

[Q9.3] If you have heard of Web-based species occurrence information systems (including any not listed in the previous question), how have you heard of those systems (e.g. from colleagues, professional meetings, peer-reviewed literature, etc.)?

[Display This Question: If 'No' is selected for Q8.3]

____

____

[Q9.4] What do you believe are the primary reasons people in your line of work do not use Web-based species occurrence information system?

[Display This Question: If 'No' is selected for Q8.3]

____

____

Your use of web-based species occurrence information systems

[Q10.1] On average, what percentage of your total use of species occurrence data in the past 12 months has come from Web-based species occurrence information systems as opposed to other media or non-Web resources?

[Display This Question: If 'Yes' is selected for Q8.3]

☐ 76 - 100 % [4]

☐ 51 - 75 % [3]

☐ 26 - 50 % [2]

☐ 0 - 25 % [1]

☐ Other. Please specify. [90] ______________________________

[Q10.2] Please list the Web-based species occurrence information system(s) you have used in the past 12 months to view or access species occurrence data. (These may include systems that provide data at any geographic scale, systems that provide data from only one source, or systems that provide data from multiple sources.)

[Display This Question: If 'Yes' is selected for Q8.3]

____

____

[Q10.3] How did you learn about the Web-based species occurrence information system(s) you use the most? Please select all that apply.

[Display This Question: If 'Yes' is selected for Q8.3]

☐ Mentors, advisers [1]

☐ Employers [1]

☐ Colleagues [1]

☐ Meetings / conferences [1]

☐ Journals, publications [1]

☐ Web search engines [1]

☐ Other. Please specify. [1] ______________________________

[Q10.4] On average, how often do you use Web-based species occurrence information systems?

[Display This Question: If ‘Yes’ is selected for Q8.3]

☐ Daily [7]

☐ Weekly [6]

☐ Monthly [5]

☐ Every 3 months [4]

☐ Every 6 months [3]

☐ Annually [2]

☐ Never or less than once per year [1]

[Q10.5] In a typical work week in which you use a Web-based species occurrence information system, how many hours (or fraction of an hour) would you say on average that you use Web-based species occurrence information systems?

[Display This Question: If ‘Yes’ is selected for Q8.3]

____

[Q10.6] Please indicate your level of use or awareness of the Web-based species occurrence information systems listed below.

[Display This Question: If ‘Yes’ is selected for Q8.3]

	Have used in the past 12 months [4]	Have used more than a year ago [3]	Have never used but have heard of it [2]	Have never heard of this resource [1]
Biodiversity Information Serving Our Nation (BISON) [Q10.6_1]	☐	☐	☐	☐
Encyclopedia of Life (EOL) [Q10.6_2]	☐	☐	☐	☐
Global Biodiversity Information Facility (GBIF) [Q10.6_3]	☐	☐	☐	☐
Integrated Digitized Biocollections (iDigBio) Portal [Q10.6_4]	☐	☐	☐	☐

	Have used in the past 12 months [4]	Have used more than a year ago [3]	Have never used but have heard of it [2]	Have never heard of this resource [1]
Map of Life (MOL) [Q10.6_5]	☐	☐	☐	☐
NatureServe Explorer [Q10.6_6]	☐	☐	☐	☐
Ocean Biogeographic Information System (OBIS) [Q10.6_7]	☐	☐	☐	☐
PLANTS Database [Q10.6_8]	☐	☐	☐	☐
VertNet [Q10.6_9]	☐	☐	☐	☐

[Q10.7] In addition to species occurrence data, are there other types of biodiversity data that you have used from Web-based information resources in your current job? (Examples of other types of biodiversity data include genetic diversity, phylogeny, life history and demographics, species traits, biological communities and species interactions, ecosystems and functional diversity, etc.)

[Display This Question: If 'Yes' is selected for Q8.3]

☐ Yes. Please specify what other types of biodiversity data you have used from Web-based resources. [2] ____

☐ No [1]

[Q10.8] How important is the availability of Web-based species occurrence information systems for the work you do?

[Display This Question: If 'Yes' is selected for Q8.3]

☐ Extremely important [7]

☐ Important [6]

☐ Somewhat important [5]

☐ Neither important nor unimportant [4]

☐ Somewhat unimportant [3]

☐ Unimportant [2]

☐ Extremely unimportant [1]

☐ No opinion or undecided [999]

[Q10.9] If you would like to elaborate on your response to the question above, feel free to do so here.

[Display This Question: If 'Yes' is selected for Q8.3]

____

____

.

[Q10.10] For the following, please rate on average the ease of use of Web-based species occurrence information systems and the data they provide based on your experience with those systems.

[Display This Question: If 'Yes' is selected for Q8.3]

	Extremely easy [7]	Easy [6]	Somewhat easy [5]	Neither easy nor difficult [4]	Somewhat difficult [3]	Difficult [2]	Extremely difficult [1]	No opinion or undecided [999]
Finding available species occurrence information systems [Q10.10_1]	☐	☐	☐	☐	☐	☐	☐	☐
Accessing species occurrence information systems [Q10.10_2]	☐	☐	☐	☐	☐	☐	☐	☐
Using species occurrence information systems [Q10.10_3]	☐	☐	☐	☐	☐	☐	☐	☐
Identifying relevant data in information systems [Q10.10_4]	☐	☐	☐	☐	☐	☐	☐	☐

	Extremely easy [7]	Easy [6]	Somewhat easy [5]	Neither easy nor difficult [4]	Somewhat difficult [3]	Difficult [2]	Extremely difficult [1]	No opinion or undecided [999]
Understanding context of the data provided by information systems [Q10.10_5]	☐	☐	☐	☐	☐	☐	☐	☐
Evaluating quality and trustworthiness of the data provided by information systems [Q10.10_6]	☐	☐	☐	☐	☐	☐	☐	☐
Retrieving data in the needed format [Q10.10_7]	☐	☐	☐	☐	☐	☐	☐	☐

[Q10.11] If you would like to elaborate on your ratings above, feel free to do so here.

[Display This Question: If ‘Yes’ is selected for Q8.3]

____

____

[Q10.12] On average, how useful are the Web-based species occurrence information systems you have used the most?

[Display This Question: If ‘Yes’ is selected for Q8.3]

☐ Extremely useful [7]

☐ Useful [6]

☐ Somewhat useful [5]

☐ Neither useful nor useless [4]

☐ Somewhat useless [3]

☐ Useless [2]

☐ Extremely useless [1]

☐ No opinion or undecided [999]

[Q10.13] What features or functions of the Web-based species occurrence information system(s) you use the most do you find most useful?

[Display This Question: If ‘Yes’ is selected for Q8.3]

[Q10.14] How could the Web-based species occurrence information systems you use the most be improved or made more useful to you?

[Display This Question: If ‘Yes’ is selected for Q8.3]

The next questions ask about end-products and/or decisions resulting from your use of Web-based species occurrence information systems and their data. Examples of end-products include scientific articles, environmental assessments, agency reports, maps, outreach materials (presentations, fact sheets), etc.

[Q10.16] What are the most common end-products and/or decisions resulting from your use of Web-based species occurrence information systems and their data?

[Display This Question: If ‘Yes’ is selected for Q8.3]

[Q10.17] Are those end-products and/or decisions available to the public?

[Display This Question: If ‘Yes’ is selected for Q8.3]

☐ Yes [2]

☐ No [1]

☐ Other. Please specify. [90] ______________________________

[Q10.18] If publicly available, where can those end-products and/or decisions be accessed? (Examples include professional journals, conference proceedings, institution public website, community websites or applications, by request via telephone, email or online forms, etc.)

[Display This Question: If ‘Yes’ is selected for Q8.3]

____

____

[Q10.19] What in your opinion has been the greatest benefit of using Web-based species occurrence information systems?

[Display This Question: If ‘Yes’ is selected for Q8.3]

____

____

[Q10.20] What in your opinion has been the greatest challenge in using Web-based species occurrence information systems?

[Display This Question: If 'Yes' is selected for Q8.3]

____

____

[Q10.21] How likely are you to use a Web-based species occurrence information system in the next 12 months?

[Display This Question: If 'Yes' is selected for Q8.3]

☐ Extremely likely [7]

☐ Likely [6]

☐ Somewhat likely [5]

☐ Neither likely nor unlikely [4]

☐ Somewhat unlikely [3]

☐ Unlikely [2]

☐ Extremely unlikely [1]

☐ No opinion or undecided [999]

End of survey

[Q11.1] You have reached the end of the survey. If you have any additional comments you would like to share about this survey or your use of species occurrence data and Web-based species occurrence information systems, please provide them here. When you are done, please click on the 'Submit' button to submit your responses and complete the survey.
