# Supplementary material for: Use of web-based species occurrence information systems by academics and government professionals
Source: PLoS One. 2020 Jul 31;15(7):e0236556. doi: 10.1371/journal.pone.0236556 (PMC7394390; doi:10.1371/journal.pone.0236556)
Supplement: S2 Appendix — (PDF) [file pone.0236556.s002.pdf]

S2 Appendix. Disciplines / fields and type of agencies searched for potential invitees to the survey.

Academic disciplines / fields relevant for this study

a) Biology and Related Fields – including agricultural science, animal science, behavior, biogeography, botany, conservation biology, ecology, ecosystem science, entomology, evolutionary biology, genetics, fisheries science, forestry, horticulture, marine biology, microbiology, molecular biology, nematology, physiology, plant pathology, quantitative biology, restoration biology, wildlife biology, zoology, and natural history museums / herbaria / botanical gardens
b) Earth, Ocean and Environmental Fields – atmospheric science, climate change research, environmental policy, environmental science, geography, geosciences (earth science, geology), hydrology, limnology, marine sciences / oceanography, natural resource studies, soil science, and sustainability studies
c) Other Fields – including environmental engineering, informatics and computing, landscape architecture, and planning

Types of government agencies relevant for this study

a) Agriculture (plant industry)

b) Conservation

c) Environmental protection (conservation, management, quality): including coastal resources, ecological resources (ecosystems, landscape), land resources, marine resources, water resources (watershed protection / stewardship)

d) Fish and wildlife (fisheries, game, nongame)

e) Fire protection

f) Forestry

g) Natural heritage (biological surveys)

h) Natural resources

i) Public lands (parks, preserves)

j) Recreation
