# Supplementary material for: Use of web-based species occurrence information systems by academics and government professionals
Source: PLoS One. 2020 Jul 31;15(7):e0236556. doi: 10.1371/journal.pone.0236556 (PMC7394390; doi:10.1371/journal.pone.0236556)
Supplement: S3 Appendix — (PDF) [file pone.0236556.s003.pdf]

S3 Appendix. Disposition codes of online survey and relevant equations.

Disposition codes

1. Returned questionnaires: 942
    a. Complete: 913
        i. Complete (from individual email): 861
        ii. Complete (from generic link): 52
    b. Partial with sufficient information: 29
        i. Partial with sufficient information (from individual email): 26
        ii. Partial with sufficient information (from generic link): 3

[Number of returned questionnaires from: individual email = 887; generic link = 55]

2. Eligible, “Non-Interview” (Non-response): 133
    a. Refusal: 118
        i. Explicit refusal (emailed the PI with reason for not participating): 22
        ii. Implicit refusal: 96
            1. Declined to participate in survey online (answer “1” to Q1.3 of survey): 24 (from individual email) (none from generic link)
            2. Opted out of survey invitation online: 66

3. Blank surveys (logged on to survey, did not complete any items): 6
    a. Blank (from individual email): 5
    b. Blank (from generic link): 1

b. Break-off with insufficient information (no answer Q3.3 and up): 11
    i. Break-off (from individual email): 10
    ii. Break-off (from generic link): 1

c. Non-contact: 4
    i. Respondent was unavailable during field period: 4

[Number of Eligible, “Non-Interview” from: individual email = 131; generic link = 2]

3. Unknown eligibility, “non-interview”: 6,156
    a. No invitation sent (failed to be sent by Qualtrics): 1
    b. Invitation returned undelivered: 110
    c. Nothing ever returned OR nothing known about respondent / email address: 6,045

4. Not Eligible, Returned: 231
    a. Screened Out of Sample (based on Q3.3 of survey): 27
        i. Screened out (from individual email): 26
        ii. Screened out (from generic link): 1

b. Other: Respondent sent email to PI indicating survey not relevant to him/her: 204
    i. Not right person to participate (reason): 45 (Adjusted final number)
    ii. No use / reuse species occurrence data (reason): 150
    iii. No use Web-bases species occurrence information systems (reason): 9

[Number of Not Eligible, Returned from: individual email = 230; generic link = 1]

Definitions of disposition code categories

a) From individual email – refers to recorded surveys in Qualtrics that were accessed from a link included in the individual email invitations that were sent to potential participants.
b) From generic link – refers to recorded surveys in Qualtrics that were accessed from the generic link sent to some survey participants who had indicated they would share the survey with more appropriate colleagues.
c) Returned questionnaires (1) – defined as survey responses (records) recorded in Qualtrics with at least sufficient data for minimal analysis.
d) Complete surveys (1a) – refer to surveys that had a progress of 100 in Qualtrics and answered at least one of the following two key questions that applied to their particular case:' Q5.3 Do you reuse species occurrence data from others in your work?'; and 'Q8.3 Have you used a Web-based species occurrence information system at least once in the past 12 months?'. If they answered at least one of these

questions even if some questions in between were not answered or skipped by the respondent, these surveys are still considered complete as minimal analysis can be done of at least the key questions.

e) Partial with sufficient information (1b) – refer to surveys that had at least a response for key question ‘Q3.3 Which of the following best describes your use of species occurrence data?’ and that had a progress somewhere between 42 to 99 percent in Qualtrics.

f) Explicit refusal (2ai) – refer to survey invitees who contacted PI via email to notify that although eligible they would not participate in the survey due to several reasons including not having time, were retired, don’t want to participate in surveys, cannot participate due to agency policies, etc.

g) Declined to participate in survey online (2aii1) – refer to surveys that answered “1” (decline to participate) to Q1.3 of survey at end of informed consent.

h) Opted out of survey invitation online (2aii2) – refers to invitees who themselves clicked on the opt-out link of Qualtrics to stop receiving notifications about the survey and who never opened or submitted a survey via Qualtrics.

i) Blank surveys (2aii3) – refer to surveys recorded in Qualtrics where the respondent answered ‘2’ agree to participate to Q1.3 after the informed consent of the survey but did not answer any subsequent questions of the survey.

j) Break-off with insufficient information (2b) - refers to surveys where respondents answered ‘2’ to Q1.3 after the informed consent, but did not answer ‘Q3.3 Which of the following best describes your use of species occurrence data?’ or any subsequent questions.

k) Respondent was unavailable during field period (2ci) - refer to survey invitees who contacted PI via email to notify they could not participate in the survey due to field season.

l) No invitation sent (3a) – Qualtrics failed to send the invitation for specific invitees. It may have been a typo or mistake in the email address.

m) Invitation returned undelivered (3b) – email invitations that bounced back in Qualtrics.

n) Nothing ever returned OR nothing known about respondent or address (3c) – Calculated from the following codes:

Accounted for invitations = Returned questionnaires (complete and partial surveys from individual email) (887) + Eligible, “Non-Interview” (from individual email) (131) + Not Eligible, Returned (from individual email) (230) + No invitation sent from Qualtrics (1) + Invitation returned undelivered from Qualtrics (110) = 1,359

Nothing known about respondent = total number of emails incorporated into Qualtrics (7,404) - number of Accounted for invitations (1,359) = 6,045

o) Screened out of sample (4a) – refer to surveys in which respondents who agreed to participate in the survey, indicated for question 3.3 a response of “1” have never used species occurrence data, and had a progress of ‘100’ or just stopped taking the survey after answering this question and nothing is known about whether or not they reuse species occurrence data from others). These surveys will be removed from the primary data analysis because they don’t use species occurrence data and therefore the survey is not relevant to them.

p) Other: Respondent sent email to PI indicating survey not relevant to him/her (4b) – refer to invitees who sent an email to PI indicating they were not participating in the survey because the survey was not relevant to them (e.g. don't use species occurrence data or reuse data from others).

Equations

Completion rate and response rate were calculated based on the disposition codes of responses from email invitations. Responses from the anonymous link were not included in these rate calculations.

Survey completion rate

$$\text{Completion Rate} = \frac{\text{Number of completed surveys}}{\text{Number of respondents who entered the survey}}$$

Or

$$\text{Completion Rate} = \frac{\text{Complete}}{\text{Complete} + \text{Partial} + \text{Blank} + \text{Break-off}}$$

$$\text{Completion Rate} = \frac{861}{861 + 26 + 5 + 10} = 0.9545$$

Proportion of unknowns that are eligible (e)

$$e = \frac{\text{Returned questionnaires} + \text{Eligible, "Non-Interview"}}{(\text{Returned questionnaires} + \text{Eligible, "Non-Interview"}) + \text{Not Eligible, Returned}}$$

$$e = \frac{887 + 131}{(887 + 131) + 230} = 0.8157$$

Survey response rate (RR4)

$$RR4 = \frac{\text{Complete} + \text{Partial}}{(\text{Complete} + \text{Partial}) + (\text{Refusal} + \text{Break-off} + \text{Non-contact}) + e(\text{Nothing known about respondent} + \text{No invitation sent} + \text{Invitation returned undelivered})}$$

$$RR4 = \frac{861 + 26}{(861 + 26) + (117 + 10 + 4) + 0.8157(6{,}045 + 1 + 110)} = 0.1469$$
